# The gap before real clinical application of imaging-based machine-learning and radiomic models for chemoradiation outcome prediction in esophageal cancer: a systematic review and meta-analysis

**DOI:** 10.1097/JS9.0000000000000441

**Published:** 2023-07-17

**Authors:** Zhi Yang, Jie Gong, Jie Li, Hongfei Sun, Yanglin Pan, Lina Zhao

**Affiliations:** a Department of Radiation Oncology, Xijing Hospital; b State Key Laboratory of Cancer Biology, National Clinical Research Center for Digestive Diseases and Xijing Hospital of Digestive Diseases, Air Force Medical University, Xi’an, People’s Republic of China

**Keywords:** chemoradiotherapy, clinical outcome, esophageal cancer, quality improvement, radiomics

## Abstract

**Background:**

Due to tumoral heterogeneity and the lack of robust biomarkers, the prediction of chemoradiotherapy response and prognosis in patients with esophageal cancer (EC) is challenging. The goal of this study was to assess the study quality and clinical value of machine learning and radiomic-based quantitative imaging studies for predicting the outcomes of EC patients after chemoradiotherapy.

**Materials and methods:**

PubMed, Embase, and Cochrane were searched for eligible articles. The methodological quality and risk of bias were evaluated using the Radiomics Quality Score (RQS), Image Biomarkers Standardization Initiative (IBSI) Guideline, and Transparent Reporting of a multivariable prediction model for Individual Prognosis or Diagnosis (TRIPOD) statement, as well as the modified Quality Assessment of Diagnostic Accuracy Studies (QUADAS-2) tool. A meta-analysis of the evidence focusing on predicting chemoradiotherapy response and outcome in EC patients was implemented.

**Results:**

Forty-six studies were eligible for qualitative synthesis. The mean RQS score was 9.07, with an adherence rate of 42.52%. The adherence rates of the TRIPOD and IBSI were 61.70 and 43.17%, respectively. Ultimately, 24 studies were included in the meta-analysis, of which 16 studies had a pooled sensitivity, specificity, and area under the curve (AUC) of 0.83 (0.76–0.89), 0.83 (0.79–0.86), and 0.84 (0.81–0.87) in neoadjuvant chemoradiotherapy datasets, as well as 0.84 (0.75–0.93), 0.89 (0.83–0.93), and 0.93 (0.90–0.95) in definitive chemoradiotherapy datasets, respectively. Moreover, radiomics could distinguish patients from the low-risk and high-risk groups with different disease-free survival (DFS) (pooled hazard ratio: 3.43, 95% CI 2.39–4.92) and overall survival (pooled hazard ratio: 2.49, 95% CI 1.91–3.25). The results of subgroup and regression analyses showed that some of the heterogeneity was explained by the combination with clinical factors, sample size, and usage of the deep learning (DL) signature.

**Conclusions:**

Noninvasive radiomics offers promising potential for optimizing treatment decision-making in EC patients. However, it is necessary to make scientific advancements in EC radiomics regarding reproducibility, clinical usefulness analysis, and open science categories. Improved model reporting of study objectives, blind assessment, and image processing steps are required to help promote real clinical applications of radiomics in EC research.

## Introduction

Highlights

- Exact patient stratification and personal treatment decisions have become an urgent necessity to improve esophageal cancer (EC) clinical outcomes.
- Due to the low methodological robustness and quality of the machine learning-based radiomic studies, it is difficult to achieve real clinical application in EC patients.
- A meta-analysis identified the promising potential for optimizing the neoadjuvant chemoradiotherapy or definitive chemoradiotherapy decision-making in EC patients.
- Room for improvement was shown in Radiomics Quality Score (RQS), Transparent Reporting of a multivariable prediction model for Individual Prognosis or Diagnosis (TRIPOD), and Image Biomarkers Standardization Initiative (IBSI) in standardized image processing steps, improved analytical methodologies, and multicenter based external validation.

Esophageal cancer (EC) is one of the most common gastrointestinal malignancies, with more than 500 000 cancer deaths per year^1^. EC principally contains two different pathological types, namely, esophageal adenocarcinoma (EAC) and squamous cell carcinoma (ESCC). To date, the 5-year survival rate of either EAC or ESCC patients is still unfavorable, with less than 25% combined all stages^2,3^. The exact patient stratification and personal treatment decision have become an urgent necessity to improve clinical outcomes.

Despite the limited therapeutic alternatives available for patients with locally advanced, unresectable, or metastatic EC, concurrent chemoradiotherapy (CCRT) serves as an essential antitumor strategy throughout the whole treatment duration regardless of neoadjuvant, curative, and palliative options^4^. In the case of resectable locally advanced EC, neoadjuvant chemoradiotherapy (NCRT) followed by surgery is considered as the standard treatment. If a pathological complete response (pCR) can be reached following NCRT, then an active surveillance and organ preservation strategy can be considered^5^. However, the pCR rate of NCRT is only 43–49% in ESCC and 23% in EAC patients^6^. Although definitive chemoradiotherapy (DCRT) serves as an optimal option for unresectable locally advanced EC, more than half of patients developed local recurrence with low survival expectancy after standard-dose DCRT^7^. Thus, realizing early identification of a complete or objective response and prediction of the risk of relapse may optimize and facilitate individualized CCRT strategies in clinical decisions. Given the invasive nature and sample limitation of molecular biomarkers derived from biopsy or surgery tissue, future research on the optimal timing of treatment sequence may benefit from the noninvasive biomarker. Nevertheless, the reliability of currently available imaging methods, including endoscopy/endoscopic ultrasound, computed tomography (CT), positron emission tomography (PET), and MR, to identify the treatment response and residual malignancy after CCRT is controversial^8,9^. Consequently, a novel approach or biomarker to help offer personalized decision-making and treatment is urgently needed.

Radiomics, a high-throughput method to extract quantitative and minable data from medical images, can produce novel imaging biomarkers that may not be visible to the naked eye^10^. Unlike invasive biopsy-based approaches, radiomics offers a noninvasive and reproducible way to obtain molecular data and is not prone to sampling bias, as it depicts the entire tumor burden. Recent machine learning-based radiomic analysis has been successfully devoted to increased accuracy in the prediction of therapy response and evaluation of prognosis^11^. Nonetheless, due to the great difference in imaging quality, processing kernel and technical setting, it is still difficult to translate radiomics into a clinically useful tool^12^.

Although radiomic analysis seems promising for the management of EC patients by predicting pathological response after NCRT^13,14^, there is still a lack of comprehensive assessment of the methodological robustness and quality of EC radiomic studies in CCRT management before their clinical translation. In addition, there has been no report to fully assess the pooled predictive power and potential clinical value of the current radiomic model regarding EC CCRT. Meaningfully, there are still disputes about whether it can actually be used in clinical practice. Hence, the purpose of our study was to assess the methodological robustness and reporting quality of radiomics studies on predicting chemoradiotherapy response or prognosis in EC patients and to support evidence for their clinical application according to the results of the meta-analysis.

## Materials and methods

This study was conducted according to the Preferred Reporting Items for Systematic Reviews and Meta-analyses (PRISMA) statement (Supplemental Digital Content 1, http://links.lww.com/JS9/A768), (Supplemental Digital Content 2, http://links.lww.com/JS9/A769), and AMSTAR-2 guidelines^15,16^ (Supplemental Digital Content 3, http://links.lww.com/JS9/A770). The study protocol was drafted and registered as CRD42022371720.

### Literature search and study selection

A comprehensive search of articles was performed in PubMed, Cochrane, and Embase databases until 31 October 2022, with a search string combining ‘esophageal neoplasms’, ‘esophageal cancer’, radiomic, textural, texture, histogram, CT, ‘computed tomography’, PET, ‘positron emission tomography’, MRI, MR, ‘magnetic resonance imaging’ and ‘magnetic resonance’. Following the exclusion of duplicates, two reviewers with a combined expertise of 5 years in radio-oncology and radiomic research carried out a basic screening of the titles and abstracts. Disagreements were resolved by consensus or with the help of a third reviewer. We included all primary researches evaluating the role of radiomic features in EC patients treated with chemoradiotherapy for predictive or prognostic purposes. The criteria for excluding studies were as follows: studies only included quantitative imaging features of volume, PET SUV, and/or MTV, without the inclusion of any additional engineered ‘radiomics’ features; studies in phantom or animal models; reviews, technical reports, letters to editors, comments, conference proceedings, case reports, and articles with insufficient information for assessing the methodological quality. Additionally, we also identified relevant articles of possible interest by screening the reference list of included studies and reviewed for eligibility. More detailed search strategies and study selection are described in Supplementary Material Note S1-2 (Supplemental Digital Content 4, http://links.lww.com/JS9/A771).

### Data extraction and quality assessment

The information, including literature information, study characteristics, and model metrics, were extracted by one reviewer from each study (Supplementary Note S3, Supplemental Digital Content 4, http://links.lww.com/JS9/A771). Then, the results were cross-examined by the other reviewer. The disagreements were solved by a third reviewer.

The eligible articles were independently evaluated by the two reviewers using the Radiomics Quality Score (RQS) checklist^17^, the modified Quality Assessment of Diagnostic Accuracy Studies (QUADAS-2) tool^18^, the Transparent Reporting of a multivariable prediction model for Individual Prognosis or Diagnosis (TRIPOD) checklist^19^ and the Image Biomarkers Standardization Initiative (IBSI) guideline^20^. The RQS was recently acknowledged as a specific radiomic tool, interrogating 16 items to estimate the methodological rigor of radiomic workflow. Recent studies assigned RQS components to six key domains that improved the integration and utilization of RQS in radiomic approaches^21^. The TRIPOD checklist, composed of 37 elements in 22 criteria, was used to measure the completeness of the relevant prediction models^19^. Previous studies have modified the TRIPOD checklist for better application in radiomic prediction models^21^. Only seven items from the IBSI guideline were chosen to evaluate the preprocessing details of eligible studies because of the overlap with the RQS and the TRIPOD^22^. The QUADAS-2 tool was adjusted to the radiomic topic via signaling questions for application concerns and risk of bias^23^. Additionally, the trial classification criteria for the proposed image mining tools^24^ and the model type category from the TRIPOD statement^25^ were used to evaluate the gap between research and clinical utilization. The detailed individual items of each guideline are described in Supplementary Tables S1–S6 (Supplemental Digital Content 4, http://links.lww.com/JS9/A771).

### Data synthesis and analysis

The RQS score and adherence rates of RQS, the TRIPOD checklist, and the IBSI guideline were calculated. If each item had a score of at least one without any deductions, it was deemed to have basic adherence, as those that have been identified^21,23^. TRIPOD adherence was calculated without items from ‘if done’, ‘if relevant’, or ‘validation’.

Two meta-analyses were implemented in the quantitative synthesis: a meta-analysis of studies investigating the use of radiomics to predict NCRT or CCRT response and another meta-analysis of articles about the utilization of radiomics to compare chemoradiotherapy outcomes such as disease-free survival (DFS) and overall survival (OS) between high-risk and low-risk groups in overall datasets. In the first meta-analysis, only papers from which two-by-two contingency tables had been collected or created using publicly available data were included. Sensitivity, specificity, positive and negative likelihood ratio, diagnostic odds ratio (DOR), and 95% CIs were calculated as the effect size. The diagnostic accuracy was exhibited as a summary receiver operating characteristic (SROC) curve. The model with the best discrimination performance, if more than one model was presented in the same research or the same cohort from different studies, was chosen to avoid duplicates. Articles with a directly documented hazard ratio (HR) and 95% CI were included in the second meta-analysis. If HRs were not reported, their estimation were calculated from published Kaplan–Meier (K–M) curves based on a previous study^26^. DFS was calculated as the interval from chemoradiation to tumor recurrence or death. OS was determined as the interval from cancer chemoradiation to death or the last follow-up. The evaluation of response to NCRT and DCRT referred to the Mandard standard and the Response Evaluation Criteria in Solid Tumors 1.1 (RECIST 1.1), respectively.

Cochran’s *Q* test and the Higgins *I* ^2^ test were utilized for heterogeneity assessment among studies. *I* ^2^ values of 0–25, 25–50, 50–75, and greater than 75% indicate insignificant, low, moderate, and high heterogeneity, respectively. Subgroup and regression analyses were further conducted to investigate the possible cause of the heterogeneity. The following sub-analyses were employed in the meta-analysis: sample size less than 100 versus greater than 100; dataset source from training or validation cohort; model classifier with LR versus SVM; imaging modalities (CECT vs. PET vs. MRI); only radiomic model versus combination with clinical factor; diverse radiomic features (combined vs. noncombined features; or radiomic vs. deep learning signatures); and treatment option (NCRT vs. DCRT), if necessary.

Statistical analysis was performed using SPSS software version 27.0, while the meta-analysis was conducted with Stata software version 15.1 with the metan, midas, metandi, and metaprop packages. Due to the presumptive variations between trials, the random effects model was chosen for calculation. A two-tailed *P*-value <0.05 was considered statistical significance unless otherwise specified. Deeks funnel plot, Egger’s, and Begg’s tests were also conducted to assess publication bias. We also used the trim and fill method to estimate the robustness of the meta-analyses.

## Results

### Literature search

**Figure 1:**
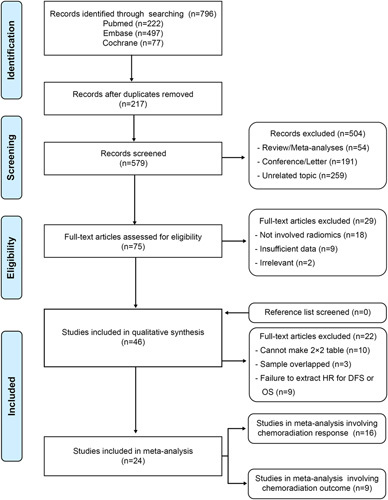
Flow diagram of study inclusion. DFS, disease-free survival; HR, hazard ratio; OS, overall survival.

The search strategy yielded 796 records in total, including 222 studies from PubMed, 497 from Embase, and 77 from Cochrane. After the removal of 217 duplicates, 579 unique records of titles and abstracts were screened, and 75 eligible articles were retrieved as full text. Ultimately, 46 articles were included in this systematic review^27–72^. By manually searching through their reference lists or relevant reviews, no other eligible studies could be identified. Eventually, 24 articles were included in the meta-analysis^49–72^, and the reasons for exclusion were as follows: ten studies failed to extract or reconstruct two-by-two tables; nine studies failed to extract HR and 95% CI or their estimation; and three studies had overlapping cohorts or datasets (Fig. 1). Notably, one study reported the predictive ability of a radiomic model on the response and outcome of chemoradiotherapy, which was included in both the first and second meta-analysis^54^.

### Study characteristics

The characteristics of the 46 included studies are presented in Table 1. The sample size of the included articles was relatively small, with a mean±deviation of 117.13±87.99, ranging from 20 to 397 patients with a median of 84 patients. All patients received chemoradiotherapy with either a neoadjuvant dose or a definitive dose.^18^ F-FDG PET/CT was the most utilized imaging modality (45.70%), followed by contrast-enhanced CT (37.00%). More than half of the models aimed to predict the response to CCRT (65.20%), followed by prognostic models for survival (23.90%) and those for recurrence (4.30%). Most studies acquired images from baseline scans (71.70%), and the other studies also extracted imaging at additional time points, including two studies during treatment and eleven studies during follow-up. Notably, only five studies addressed deep learning-based radiomics. Most models (36.50%) were verified using the same cohort with or without resampling, whereas just a few (21.70%) were validated externally. According to the phase classification criteria for image mining studies, 13 studies (28.70%) were identified as phase 0, followed by 12 studies as phase II (26.10%), and 12 studies as discovery science (26.10%). The detailed characteristics of individual studies are presented in Table 2 and Supplementary Table S7 (Supplemental Digital Content 4, http://links.lww.com/JS9/A771).

**Table 1:** Characteristics of included studies

Study characteristics	Data
Sample size, mean±SD, median (range)	117.13±87.99, 84 (20–397)
Biomarker, *n* (%)	*N*=46
Predictive	30 (65.20)
Prognostic	13 (28.20)
Predictive+Prognostic	3 (6.50)
Imaging modality, *n* (%)	*N*=46
CT	17 (37.00)
MRI	7 (15.20)
PET	21 (45.70)
PET+CT	1 (2.20)
Imaging temporal order	*N*=46
Pretreatment	33 (71.70)
Pretreatment+Mid-treatment	2 (4.30)
Pretreatment+Post-treatment	11 (23.90)
Model algorithm	*N*=46
Machine learning	41 (89.13)
Deep learning	5 (10.87)
Model type, *n* (%)	*N*=46
Type 1a: Developed model evaluated with exactly the same data	12 (26.10)
Type 1b: Developed model validated with resampling data	14 (30.40)
Type 2a: Developed model validated with randomly splitting data	10 (21.70)
Type 2b: Developed model validated with non-randomly splitting data	0 (0.00)
Type 3: Developed model validated with separate data	10 (21.70)
Type 4: Validation only	0 (0.00)
Phase classification, *n* (%)^a^	*N*=46
Discovery science: experimental	12 (26.10)
Phase 0:<100 patients; retrospective; internal validation	13 (28.30)
Phase I:<100 patients; retrospective; external validation	5 (10.90)
Phase II:>100 patients; retrospective; external validation	12 (26.10)
Phase III:>100 patients; prospective; external validation	4 (8.70)
Phase IV:real-world	0 (0.00)

a Two studies were classified as phase III due to prospective research, although they were conducted with less than 100 patients. Three studies were classified as phase 0 due to internal validation, although they were conducted with more than 100 patients.

**Table 2:** PRISMA literature list

References	Treatment	Study design	Nonradiomics information	Validation	Extraction software	Segmentation	Imaging features	Target condition definition	Reference standard	Classifier
Beukinga 2022	Five cycles of carboplatin and paclitaxel) with 41.4 Gy in 23 fractions	Retrospective single-center	None	Cross-validation	Matlab	Manual	Radiomic texture features	Response to nCRT	Histopathology	LASSO, logistic regression; SVM; NB; KNN
Tang 2021	NA	Retrospective single-center	Clinical information	Split sample	IBEX MATLAB	Manual	Shape features+GLCM+GLRLM features+Intensity histogram	Recurrence	Follow-up; Recurrence	LASSO
Murakami 2021	40Gy + 5-FU and cisplatin	Retrospective single-center	None	Cross-validation	Pyradiomics	Manual	GLCM+GLRLM+GLSZM+GLDM features	Response to nCRT	Histopathology	LASSO, NN
Ji 2021	50.0 to 60.0 Gy+cisplatin	Prospective single-center	Vascular permeabilityparameters	None	OmniKinetics	Manual	Texture parameters	Response to nCRT	Histopathology	LR
Tang 2021b	NA	Retrospective single-center	Clinical information	Split sample	IBEX MATLAB	Manual	GLCM+GLRCM features+Intensity histogram	Recurrence	Follow-up; Recurrence	LASSO
Xie 2021	40 to 44 Gy+cisplatin and vinorelbine/docetaxel; 40 to 41.4 Gy+paclitaxel and carboplatin (or cisplatin and 5-FU)	Retrospective multiple-center	Clinical information; Genomic data	External validation	PyRadiomics	Manual	First-order class+GLCM+GLDM+ GLRLM+ GLSZM features	Survival	Follow-up	LASSO
Li 2021a	40Gy/41.4 Gy/50.4Gy+PF/TC/TP/SP/NP/DP	Retrospective single-center	Clinical information	Bootstrapping validation	IBEX MATLAB	Manual	Shape features+ First-order features+ GLCM+GLRCM+Intensity histogram	Response to nCRT	Histopathology	LASSO; LR
Rishi 2021	45-56Gy +cisplatin and 5-FU	Retrospective single-center	None	Cross-validation	Mirada RTx	Semiautomatic+ Manual	Eccentricity+ LRLGE+gray-level normalized HIE+SDlac2+SDfd+ PET parameters	Response to nCRT Survival	Histopathology; OS, DFS, LRC	LR
Beukinga 2021	41.4 Gy+carboplatin and paclitaxel	Retrospective single-center	Scoring of biological tumor markers	Bootstrapping validation	Matlab 2018a	Manual	Inverse variance+coarseness+Moran’s I index+second measure of information correlation+elongation+ GLCM+ GLDZM	Response to nCRT	Histopathology	LR
Hu 2021	40 to 44 Gy+cisplatin and vinorelbine/docetaxel; 40 to 41.4 Gy+paclitaxel and carboplatin (or cisplatin and 5-FU)	Retrospective multiple-center	None	External validation	ITK-SNAP; PyRadiomics	Manual	DL features+First-order+GLDM+ GLRLM+ GLSZM+ GLCM	Response to nCRT	Histopathology	SVM; CNN; LR; RF; XG
Hu 2020	40 to 44 Gy+cisplatin and vinorelbine/docetaxel; 40 to 41.4 Gy+paclitaxel and carboplatin (or cisplatin and 5-FU)	Retrospective multiple-center	None	External validation	ITK-SNAP; PyRadiomics	Manual	First-order+ GLCM+ GLSZM features	Response to nCRT	Histopathology	Linear regression; SVM; KNN; NB; DT; RF; EGB
Chen 2019	44-63Gy+Cisplatin/5-FU (Cisplatin)	Retrospective single-center	Clinical information	Split sample	PMOD 4.0; MATLAB 2012a	Semiautomatic+ Manual	Semi-quantitative PET parameters+ NGLCM+ TFCCM+ NGTDM	Response to nCRT	Histopathology	LR
Yang 2019	40-64 Gy+ NP/PF/TP	Retrospective single-center	None	Split sample	3D Slicer	Manual	Shape features+ First-order features+ GLRLM+ GLSZM+ GLCM	Response to nCRT	Histopathology	LASSO; LR
Larue 2018	41.4 Gy+carboplatin and paclitaxel	Retrospective multiple-center	None	External validation	OncoRadiomics	Manual	GLCM+ GLDZM+ GLRLM+ NGLDM	Survival	3-year overall survival	RF
Riyahi 2018	50.4 Gy+cisplatin and 5-FU	Retrospective single-center	None	Cross-validation	NA	Automatic	Jacobian features+ GLCM+GLRM	Response to nCRT	Histopathology	LASSO; SVM
Beukinga 2018	41.4 Gy+carboplatin and paclitaxel	Retrospective single-center	None	Bootstrapping validation	Matlab 2014b	Manual	Shape features+ GLCM+ GLRLM	Response to nCRT	Histopathology	LR
Beukinga 2017	41.4 Gy+carboplatin and paclitaxel	Retrospective single-center	Clinical information	Bootstrapping validation	NA	Manual	Geometry features+first-order+GLCM +GLSZM+GLRLM	Response to nCRT	Histopathology	LR
Yip 2016	45-50.4 Gy + cisplatin and 5-FU/irinotecan/paclitaxel	Retrospective single-center	None	None	MATLAB-CGITA	Manual	GLCM+SRHGRE+HGRE+SZHGE+HGZE	Response to nCRT	Histopathology	NA
van Rossum 2016	45/50.4 Gy + Oxaliplatin/5-fluorouracil or Docetaxel/5-fluorouracil	Retrospective single-center	Clinical information	Bootstrapping validation	IBEX MATLAB	Semiautomatic	Semi-quantitative PET parameters+ geometry and first- and second-order texture features	Response to nCRT	Histopathology	LR
Yip 2016b	NA	Retrospective single-center	None	None	MATLAB-CGITA	Manual	GLCM+RLM+SZM	Response to nCRT	Histopathology	NA
Zhang 2014	50.4 Gy+cisplatin and 5-FU	Retrospective single-center	Clinical parameters	Cross-validation	NA	Semiautomatic	Semi-quantitative PET parameters+ Inertia+Energy+Entropy+Skewness	Response to nCRT	Histopathology	SVM; LR
Tan 2013	50.4 Gy+cisplatin and 5-FU	Retrospective single-center	None	None	3D slicer	Manual	Semi-quantitative PET parameters+texture features+histogram distance	Response to nCRT	Histopathology	NA
Tan 2013b	50.4 Gy+cisplatin and 5-FU	Retrospective single-center	None	None	ITK-SNAP	Semiautomatic	Semi-quantitative PET parameters+texture features+ intensity features+ Geometry features	Response to nCRT	Histopathology	NA
Hirata 2020	40Gy +cisplatin and 5-FU	Retrospective single-center	None	None	NA	Manual	ADC+histogram parameters+ kurtosis+ skewness	Response to nCRT; Survival	Histopathology; DSS, RFS	Cox
Gong 2022	50-60 Gy+ Fluorouracil/ capecitabine and cisplatin (PF) or capecitabine or S1	Retrospective multiple-center	Clinical information	Split sample+ external	Pyradiomics	Manual	Shape features+ GLCM+GLDM features+ DL network	Survival	Follow-up (LRFS, OS)	Lasso Cox DL
Jayaprakasam 2022	Induction Chemotherapy+CCRT	Retrospective single-center	None	Split sample	MATLAB	Manual+ automatic	First-order class+GLCM+NGLDM+RLM features	T/N category; Survival	AJCC; Follow-up (PFS, 3-year OS)	Ridge and LASSO regression; SVM
An 2022	50.4-66.0 Gy+paclitaxel+cisplatin/S-1+cisplatin	Retrospective multiple-center	Clinical information	Split sample+ external	3D Slicer	Manual + semiautomatic	Whole-tumor ADC values and radiomics features	Response to CCRT	RECIST1.1	SVM; LR
Luo 2021	50-72 Gy+TP/PF	Retrospective single-center	Clinical information	Split sample	3D Slicer	Manual	First-order class+texture features	Survival	Follow-up (LRFS)	Lasso Cox; Cox regression
Kong 2021	50-66Gy+FP/TP	Retrospective single-center	Clinical information	Split sample	Pyradiomics	Manual	First-order class+GLCM+GLDM+GLSZM features	Survival	Follow-up (LRFS)	Lasso Cox
Li 2021b	50.4/59.4Gy+teggio+ cisplatin	Prospective multiple-center	Clinical information	External	Monaco	Manual	DL network	Response to CCRT	RECIST1.1	CNN; DL
Luo 2020	50-72Gy+TP/PF	Retrospective single-center	Clinical information	Split sample	3D Slicer; Pyradiomics	Manual	Shape features+ NGTDM+GLDM features	Response to CCRT	Imaging criteria	LASSO logistic regression
Li 2020	50-66 Gy+DP/DF	Retrospective multiple-center	None	Split sample+ external	PyRadiomics	Automatic	Shape features+ First-order features+ GLCM+ Semi-quantitative PET parameters	Survival	Follow-up (DFS, OS, LC)	LASSO Cox regression
Cao 2020	>50Gy+DF	Retrospective multiple-center	None	External	PyRadiomics	Manual	First-order class+GLCM+GLRLM features	Response to CCRT	RECIST1.1	LASSO logistic regression
Xu 2020	60Gy+DP	Retrospective single-center	None	None	OmniKinetics	Manual	Histogram parameters	Response to CCRT; Survival	RECIST1.1; Follow-up (PFS)	Kaplan–Meier
Xie 2020	60-66Gy+Taxol plus cisplatin/Docetaxel plus cisplatin	Retrospective single-center	None	None	IBEX	Manual	Whole-tumor textural features	Survival	Follow-up (OS)	Kaplan–Meier; Cox model
Li 2019	50-63Gy+chemotherapy	Prospective single-center	None	Cross-validation	MATLAB	Manual + semiautomatic	Histogram texture+ GLCM+ GLGCM+ISZF features	Survival	Follow-up (OS)	LASSO regression; Cox regression
Sun 2019	60Gy+DP	Retrospective single-center	None	None	Omnikinetics	Manual + semiautomatic	Histogram parameters of ADC	Response to CCRT	RECIST1.1	General model for combining pairs of texture parameters
Xie 2019	54-60 Gy+ cisplatin	Retrospective multiple-center	None	External	ITK-SNAP; MATLAB 2015b	Manual	Histogram features+GLCM+ GLSZM features	Survival	Follow-up (OS)	LASSO Cox regression
Jin 2019	40-70Gy+DF	Retrospective single-center	Dosimetric parameters	Cross-validation	MATLAB	Manual	Texture features	Response to CCRT	Imaging criteria	SVM; XGBoost
Xiong 2018	67-73Gy+DF	Retrospective single-center	None	Cross- validation	MATLAB	Manual	First-order class+ textural features+ wavelet features	Local control	Follow-up	RF; SVM; LR; ELM
Hou 2018	60Gy+nedaplatin plus docetaxel/paclitaxel	Retrospective single-center	None	Split sample	IBEX MATLAB	Semiautomatica	Histogram features+ GLCM+ GLRLM+GWTF features	Response to CCRT	RECIST1.1	SVM; ANN
Hou 2017	60Gy+nedaplatin plus docetaxel/paclitaxel	Retrospective single-center	None	Split sample	MATLAB 2015a	Manual	Histogram2D+GLSZM+Gabor2D	Response to CCRT	RECIST1.1	SVM; ANN
Paul 2017	NA	Retrospective single-center	Clinical information	Cross-validation	NA	Manual	First-order+GLCM+ GLSZM+GDLM features	Response to CCRT; Survival	Imaging criteria; Follow-up	GARF
Nakajo 2016	41.4 -70 Gy +cisplatin/5-fluorouracil with or without docetaxel	Retrospective single-center	None	NA	NA	Manual+ automatic	Semi-quantitative PET parameters+ entropy, homogeneity and dissimilarity features	Response to CCRT; Survival	RECIST1.1; Follow-up	NA
Hatt 2013	60Gy+5-fluorouracil/cisplatin	Retrospective single-center	None	NA	Medcalc	Automatic	Semi-quantitative PET parameters+ heterogeneity quantification	Response to CCRT	RECIST1.1	NA
Tixier 2011	60Gy+5-fluorouracil-cisplatin or 5-fluorouracil-carboplatin	Retrospective single-center	None	NA	NA	Automatic	Semi-quantitative PET parameters+ heterogeneity quantification	Response to CCRT	RECIST1.1	NA

Bold studies included in the meta-analysis.

CNN, convolutional neural network; CT, computed tomography; DCE, dynamic contrast-enhanced; DL, deep learning; DT, decision tree; DWI diffusion-weighted image; EAC, esophageal adenocarcinoma; EC, esophageal cancer; EGB, extreme gradient boosting; ESCC, esophageal squamous cell carcinoma; GLCM, Gray-Level Co-occurrence Matrix Features; GLDM, Gray-Level Dependence Matrix Features; GLRLM, Gray-Level Run Length Matrix Features; GLSZM, Gray LevelSize Zone Matrix Features; HGRE, high-gray-run emphasis; HGZE, high-gray zone-run emphasis; KNN, k-nearest neighbors; LR, logistic regression; NB, naive bayes; nCRT, neoadjuvant chemoradiotherapy; NGLCM normalized gray-level co-occurrence matrix; NGTDM neighborhood gray-tone difference matrix; NN, neural network; OS, overall survival; RF, random forest; PET, 18F-fluorodeoxyglucose positron emission tomography; RF, random forest; RFS, relapse free survival; SRHGRE, Short-run high-gray-run emphasis; SVM, support vector machine; SZHGE, short-zone high-gray-run emphasis; TFCCM texture feature coding co-occurrence matrix; XG, XGboost Classifier.

### Quality analysis

**Figure 2:**
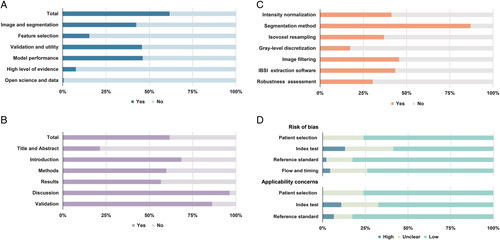
Quality assessment of the included studies. (A) Ideal percentage of Radiomics Quality Score. (B) Transparent Reporting of a multivariable prediction model for Individual Prognosis or Diagnosis adherence rate. (C) Adherence rate of Image Biomarkers Standardization Initiative preprocessing steps. (D) Quality Assessment of Diagnostic Accuracy Studies-2 assessment result.

The summered mean±SD of the RQS rating was 9.02±7.45, with an overall adherence rate of 42.52% (313/736), and an ideal percentage of RQS of 25.11% (9.04/36) (Table 3; Fig. 2A). All six essential domains showed a suboptimal percentage of an ideal score less than half percent, among which the highest ideal percentage was identified in the model performance index domain (46.23%) and the lowest percentage in the open science domain (0.54%). Although most pieces of literature provided well-documented image acquisition protocols (97.83%), none of these studies conducted phantom and cost-effectiveness analyses. Only a limited number of studies (4/46) prospectively validated radiomic biomarkers, and another four studies discussed the relevance between radiomic features and tumor biology. Seventeen studies combined clinical information, including age, TNM stage, differentiation degree, or blood tests, with radiomic models, among which thirteen reports indicated the improved predictive performance of the models after feature combination. More than half of the studies performed validation of radiomic signatures (70.29%), and 10 (21.74%) employed external cohorts from other institutes. In terms of clinical utility, only seven studies used decision curve analysis to determine if their models were ready for clinical practice.

**Table 3:** RQS rating of included studies

RQS items according to six key domains	Range	Percentage of ideal score, mean (%)	Basic adherence rate, *n* (%)
Total 16 items	−8–36	9.02 (25.06)	313/736 (42.52)
Domain 1: Protocol quality and stability in image and segmentation	0–5	2.10 (42.02)	97/184 (52.71)
1. Protocol quality	0–2	0.98 (48.91)	45/46 (97.83)
2. Multiple segmentations	0–1	0.67 (66.67)	30/46 (66.67)
3. Phantom study	0–1	0.00 (0.00)	0/46 (0.00)
4. Test–retest	0–1	0.46 (45.65)	21/46 (45.65)
Domain 2: Feature selection and validation	−8–8	1.23 (15.40)	62/92 (67.39)
5. Feature reduction or adjustment of multiple testing	−3–3	0.96 (31.88)	30/46 (65.94)
12. Validation	−5–5	0.28 (5.5)	32/46 (70.29)
Domain 3: Biologic/clinical validation and utility	0–6	2.75 (45.77)	73/184 (39.67)
6. Nonradiomics features	0–1	0.38 (37.68)	17/46 (37.68)
7. Biologic correlations	0–1	0.09 (9.42)	4/46 (9.42)
13. Comparison with ‘gold standard’	0–2	1.96 (97.82)	45/46 (97.82)
14. Potential clinical utility	0–2	0.32 (15.94)	7/46 (15.94)
Domain 4: Model performance index	0–5	2.31 (46.23)	76/138 (55.07)
8. Cutoff analysis	0–1	0.42 (42.03)	19/46 (42.03)
9. Discrimination statistics	0–2	1.54 (76.81)	44/46 (95.65)
10. Calibration statistics	0–2	0.36 (17.75)	13/46 (28.26)
Domain 5: High level of evidence	0–8	0.61 (7.61)	4/92 (4.35)
11. Prospective study	0–7	0.61 (8.70)	4/46 (8.70)
15. Cost-effectiveness analysis	0–1	0.00 (0.00)	0/46 (0.00)
Domain 6: Open science and data	0–4	0.02 (0.54)	1/46 (2.17)

RQS, Radiomics Quality Score.

Excluding items containing ‘if done’, ‘if relevant’, and ‘validation’, there was an overall adherence rate of 61.70% (795/1288) to the TRIPOD checklist (Table 4; Fig. 2B). The highest adherence rate was identified in the ‘Discussion’ section (96.38%), while the ‘Title and Abstract’ obtained the lowest adherence rate of only 21.38%. Furthermore, studies seldom followed the reports of blindness in assessments (item 6b, 4.35%), sample size calculation (item 8, 7.97%), handling of missing data (item 9, 15.21%), and explanation of model utility (item 15b, 17%).

**Table 4:** Individual TRIPOD items adherence of included studies

37 selected items in 22 criteria according to 7 sections (*N*=46)	Study adherence rate, *n* (%)
Total	795/1288 (61.70)
Section 1: Title and abstract	20/92 (21.38)
1. Title—identify developing/validating a model, target population, and the outcome	7/46 (14.49)
2. Abstract—provide a summary of objectives, study design, setting, participants, sample size, predictors, outcome, statistical analysis, results, and conclusions	12/46 (26.09)
Section 2: Introduction	63/92 (68.48)
3a. Background—explain the medical context and rationale for developing/validating the model	46/46 (100.00)
3b. Objective—specify the objectives, including whether the study describes the development/validation of the model or both	17/46 (36.96)
Section 3: Methods	358/598 (59.81)
4a. Source of data—describe the study design or source of data	46/46 (100.00)
4b. Source of data—specify the key dates	46/46 (100.00)
5a. Participants—specify key elements of the study setting including number and location of centers	46/46 (100.00)
5b. Participants—describe eligibility criteria for participants	29/46 (63.04)
5c. Participants—give details of treatment received, if relevant (*N*=46)	40/46 (86.96)
6a. Outcome—clearly define the outcome, including how and when assessed	40/46 (86.96)
6b. Outcome—report any actions to blind assessment of the outcome	2/46 (4.35)
7a. Predictors—clearly define all predictors, including how and when assessed	38/46 (83.33)
7b. Predictors—report any actions to blind assessment of predictors for the outcome and other predictors	11/46 (23.91)
8. Sample size—explain how the study size was arrived at	4/46 (7.97)
9. Missing data—describe how missing data were handled with details of any imputation method	7/46 (15.21)
10a. Statistical analysis methods—describe how predictors were handled	46/46 (100.00)
10b. Statistical analysis methods—specify type of model, all model-building procedures and method for internal validation	30/46 (64.49)
10d. Statistical analysis methods—discrimination and calibration	13/46 (28.26)
11. Risk groups—provide details on how risk groups were created, if done (*N* =15)	15/46 (32.61)
Section 4: Results	156/276 (56.64)
13a. Participants—describe the flow of participants, including the number of participants with and without the outcome. A diagram may be helpful	13/46 (27.54)
13b. Participants—describe the characteristics of the participants, including the number of participants with missing data for predictors and outcome	40/46 (87. 68)
14a. Model development—specify the number of participants and outcome events in each analysis	29/46 (63.04)
14b. Model development—report the unadjusted association between each candidate predictor and outcome, if done (*N*=19)	19/46 (40.58)
15a. Model specification—present the full prediction model to allow predictions for individuals (regression coefficients, intercept)	23/46 (50.72)
15b. Model specification—explain how to the use the prediction model (nomogram, calculator, etc.)	7/46 (15.22)
16. Model performance-report performance measures (with confidence intervals) for the prediction model	44/46 (95.65)
Section 5: Discussion	133/138 (96.38)
18. Limitations—discuss any limitations of the study	45/46 (97.83)
19b. Interpretation—give an overall interpretation of the results	46/46 (100.00)
20. Implications—discuss the potential clinical use of the model and implications for future research	46/46 (100.00)
Section 6: Other information	55/92 (59.05)
21. Supplementary information—provide information about the availability of supplementary resources, such as study	31/46 (67.39)
22. Funding—give the source of funding and the role of the funders for the present study	24/46 (52.17)
Section 7: Validation for model type 2a, 2b, 3, and 4 (*N*=20)	69/80 (86.25)
10c. Statistical analysis methods—describe how the predictions were calculated	20/20 (100.00)
10e. Statistical analysis methods—describe any model updating (recalibration), if done (*N*=0)	NA
12. Development versus validation—identify any differences from the development data in setting, eligibility criteria, outcome, and predictors	15/20 (75.00)
13c. Participants (for validation)—show a comparison with the development data of the distribution of important variables	15/20 (75.00)
17. Model updating—report the results from any model updating, if done (*N*=0)	NA
19a. Interpretation (for validation)—discuss the results with reference to performance in the development data and any other validation data	18/20 (90.00)

TRIPOD, Transparent Reporting of a Multivariable Prediction Model for Individual Prognosis or Diagnosis.

The total compliance rate of IBSI preprocessing steps was 43.17% (129/322) (Fig. 2C). The software applied for feature extraction differed among studies, including IBEX (7/46), Pyradiomics (9/46), MATLAB (12/46), and others. Among these, IBEX and PyRadiomics had IBSI compliance. Multiple segmentation methods were reported in these studies, such as manual segmentation (31/46), semiautomatic segmentation (4/46), and automatic segmentation (4/46). Moreover, the utilization of two segmentation methods was also reported in the other eight studies. Imaging filtering (45.62%) was the most commonly conducted proprocessing step, followed by intensity normalization (41.30%) and image interpolation (36.95%). Importantly, a limited number of studies reported the robustness assessment (30.43%) or gray-level discretization process (17.39%).

The results of the QUADAS-2 assessment are summarized in Figure 2D. A low-risk of bias was found in most of the included studies. Due to incomplete reporting of feature selection and a lack of external validation, the high-risk of bias and application concerns regarding index testing were commonly identified. Several studies did not provide strict eligibility criteria and enough results per predictor variable to provide estimates; thus, the corresponding risk of bias was unclear. Individual assessments per study regarding every quality analysis are summarized in Supplementary Tables S8–S11 (Supplemental Digital Content 4, http://links.lww.com/JS9/A771).

### Clinical value of radiomics in predicting chemoradiation response and outcome

**Figure 3:**
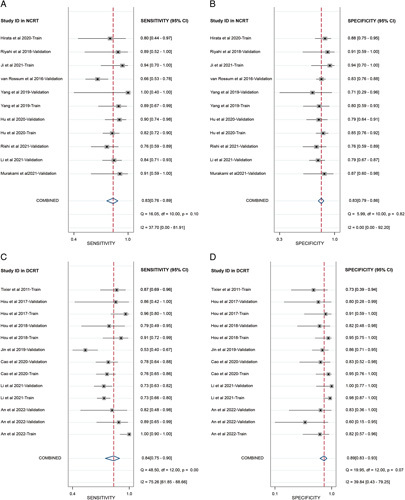
Forest plots of pooled sensitivity and specificity of imaging-based radiomics in predicting esophageal cancer concurrent chemoradiotherapy response. (A) Sensitivity in neoadjuvant chemoradiotherapy. (B) Specificity in neoadjuvant chemoradiotherapy. (C) Sensitivity in definitive chemoradiotherapy. (D) Specificity in definitive chemoradiotherapy. The numbers are pooled estimates with 95% CIs in parentheses; horizontal lines indicate 95% CIs; pooled result for all studies is presented as a black diamond. DCRT, definitive chemoradiotherapy; NCRT, neoadjuvant chemoradiotherapy.

**Figure 4:**
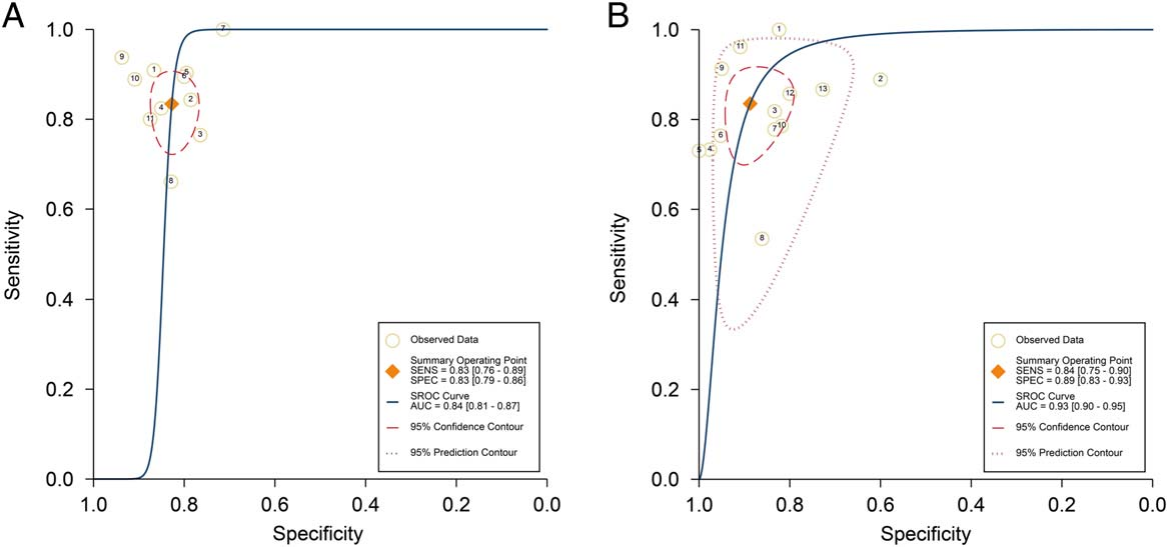
Summary receiver operating characteristic (SROC) curve of the model performance for radiomics in chemoradiotherapy response prediction. (A) SROC curve of the model performance in neoadjuvant chemoradiotherapy datasets; (B) SROC curve of the model performance in definitive chemoradiotherapy datasets. SROC, summary receiver operating characteristic.

Nine NCRT-related studies with eleven datasets (*n*=571) and seven DCRT-related studies with thirteen datasets (*n*=810) concerning response prediction were included in the first meta-analysis (Supplementary Table S12, Supplemental Digital Content 4, http://links.lww.com/JS9/A771). The results exhibited that the pooled sensitivity and specificity were 0.83 (0.76–0.89) and 0.83 (0.79–0.86), respectively, with an area under the curve (AUC) of 0.84 (0.81–0.87) for the NCRT datasets. In addition, the pooled sensitivity and specificity among the DCRT datasets were 0.84 (0.75–0.90) and 0.89 (00.83–0.93), respectively, with a satisfactory AUC of 0.93 (0.90–0.95) (Table 5 and Figs. 3–4). As well, the overall PLR, NLR, and DOR indicated their favorable predictive performance (Supplementary Figs S1 and S2, Supplemental Digital Content 4, http://links.lww.com/JS9/A771).

**Table 5:** Summary estimate of subgroup analysis in image-based radiomics in DCRT response prediction.

		Sensitivity				Specificity			
	Number of dataset	Sensitivity	*P* ^*^	*I* ^2^ (%)	*P* ^†^	Specificity	*P* ^*^	*I* ^2^ (%)	*P* ^†^
Overrall	13	0.84 (0.75–0.90)	<0.05	75.26		0.89 (0.83–0.93)	0.07	39.84	
Sample					0.06				<0.05
<100	11	0.85 (0.75–0.93)	<0.05	80.00		0.88 (0.81–0.93)	0.67	0.00	
≥100	2	0.73 (0.68–0.79)	–	–		0.99 (0.93–1.00)	–	–	
Cohort					0.68				0.44
Train	6	0.89 (0.77–0.97)	<0.05	84.93		0.92 (0.84–0.98)	0.18	34.64	
Validation	7	0.75 (0.65–0.84)	<0.05	54.30		0.88 (0.78–0.96)	0.32	14.62	
Imaging feature					0.12				<0.05
Radiomic features	7	0.83 (0.68–0.94)	<0.05	85.90		0.86 (0.78–0.93)	0.51	0.00	
DL features	6	0.82 (0.73–0.90)	<0.05	60.07		0.96 (0.89–1.00)	0.32	15.09	
Imaging modality					0.09				0.60
CECT	5	0.76 (0.63–0.87)	<0.05	80.37		0.95 (0.86–1.00)	0.18	36.33	
PET	3	0.79 (0.72–0.85)	–	–		0.87 (0.70–0.98)	–	–	
MRI	5	0.92 (0.79–0.99)	<0.05	63.76		0.87 (0.76–0.95)	0.41	0.00	
Combine model					<0.05				0.23
Only radiomic	8	0.85 (0.79–0.90)	0.27	19.67		0.88 (0.80–0.95)	0.41	2.66	
radiomic+clinical	5	0.79 (0.62–0.92)	<0.05	90.27		0.93 (0.84–0.99)	0.10	48.28	

* Note: *P*-value for heterogeneity within each subgroup.

† *P*-value for heterogeneity within meta-regression between subgroups.

**Figure 5:**
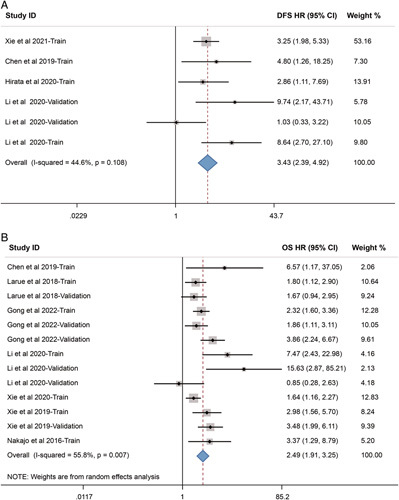
Forest plots of the predictive performance of radiomics in (A) disease-free survival and (B) overall survival of patients treated with concurrent chemoradiotherapy. Hazard ratio for each dataset is presented as a black dot, with the horizontal line indicating the 95% CI. The pooled result for all studies is presented as a black diamond. DFS, disease-free survival; HR, hazard ratio; OS, overall survival.

Four studies with six datasets (*n*=314) and seven studies with thirteen datasets (*n*=1106) concerning DFS and OS risk stratification were included in the second meta-analysis. The pooled results demonstrated that radiomic models could be used to divide the patients into high-risk and low-risk groups. The meta-analysis comparing CCRT outcomes between the two groups exhibited a pooled HR of 3.43 (95% CI 2.39–4.92) for DFS and 2.49 (95% CI 1.91–3.25) for OS (Fig. 5A and B).

### Subgroup and heterogeneity analysis

The first meta-analysis showed significant heterogeneity in sensitivity (*I* ^2^=75.26%, *P*<0.05) and low heterogeneity in specificity (*I* ^2^=39.84%, *P*=0.07) among DCRT datasets. The results of subgroup and meta-regression studies indicated that the cause of inter-study heterogeneity may be attributed to the combination with clinical factors in sensitivity analysis, while sample size and usage of the deep learning (DL) signature in specificity analysis (Table 5 and Supplementary Fig. S3, Supplemental Digital Content 4, http://links.lww.com/JS9/A771). Moreover, although there was low heterogeneity in sensitivity (*I* ^2^=37.70%, *P*=0.10) and insignificant heterogeneity in specificity (*I* ^2^=0.00%, *P*=0.82) among NCRT datasets, subgroup analysis was also performed to explore the pooled results with different study covariates (Supplementary Table S13, Supplemental Digital Content 4, http://links.lww.com/JS9/A771 and Supplementary Fig. S4, Supplemental Digital Content 4, http://links.lww.com/JS9/A771).

Datasets using the training cohort achieve higher sensitivity and specificity than those with the validation cohort in whole datasets. It seemed that the sensitivity of datasets with a sample size less than 100 was higher than that with a sample size greater than or equal to 100 in both the DCRT and NCRT datasets. Compared with CECT and PET, MRI achieved the highest sensitivity and specificity in both the DCRT and NCRT datasets. Studies using only radiomic models had higher sensitivity (0.85 vs. 0.79) but lower specificity (0.88 vs. 0.93) than studies combined with clinical models in the DCRT group. The higher sensitivity and equivalent specificity of only the radiomic predictor could be observed among the NCRT datasets. Seven DCRT datasets using normal radiomic features had an equivalent sensitivity (0.83 vs. 0.82) but lower specificity (0.86 vs. 0.96) compared to six studies using deep learning features. Moreover, the impact of different radiomic features in NCRT datasets was analyzed. Four datasets utilizing combined signatures containing morphology, first-order, and texture features showed the lowest sensitivity and specificity among datasets utilizing only first-order or texture feature or their combination.

Concerning the second meta-analysis, the *I* ^2^ statistic indicated low heterogeneity among DFS-related datasets (*I* ^2^=44.6%, *P*=0.108), while moderate heterogeneity among OS-related datasets (*I* ^2^=55.8%, *P*=0.007). Due to the small number of datasets and low heterogeneity in DFS-related analysis, the meta-regression and subgroup analysis were only performed in OS-related datasets. However, meta-regression using the above study characteristics failed to identify the source of heterogeneity. Furthermore, subgroup analysis indicated no significant changes in the pooled results for prognostic risk prediction (Supplementary Table S14, Supplemental Digital Content 4, http://links.lww.com/JS9/A771).

Moreover, a low likelihood of publication bias has been identified for these studies. Deeks’ funnel asymmetry test showed *P*>0.05 in the first meta-analysis. Egger’s and Begg’s tests showed both *P*>0.05 in the second meta-analysis (Supplementary Figs S5 and S7, Supplemental Digital Content 4, http://links.lww.com/JS9/A771). Although the trim and fill analysis showed the missing datasets, the adjusted predictive performance was still statistically significant (Supplementary Figs S6 and S8, Supplemental Digital Content 4, http://links.lww.com/JS9/A771).

## Discussion

The present study found that radiomics studies regarding EC generally lacked adequate methodological and reporting quality, as measured by the RQS, TRIPOD, and IBSI guidelines, with average overall adherence rates of 42.52, 61.70, and 43.17%, respectively. The QUADAS-2 analysis suggested a significant bias risk and application concern involving index testing. All these quality assessments may provide better guidance for future investigations. More importantly, our meta-analysis quantitatively integrated different independent data from EC chemoradiotherapy datasets and could provide essential clues for radiomic clinical value in predicting both chemoradiation response and prognosis in EC. The performance of different sample sizes, dataset sources, model classifier, imaging modalities, and diverse radiomic features or signatures were also described in our study. Potential sources of heterogeneity were determined based on the above subgroup and meta-regression analyses. Despite the promising predictive performance, their levels of evidence were all recognized as weak, suggesting that radiomics methods were far from useful tools in clinical practice.

A previous study from Kao and Hsu^14^ highlighted the clinical value of pretreatment imaging-based radiomics in predicting pCR by achieving a pooled value of 0.81 (95% CI 0.76–0.87) in EC patients who underwent NCRT. Similarly, our study exhibited a satisfying pooled AUC (0.84, 95% CI 0.81–0.87) of the included articles regarding NCRT response prediction with a larger sample size and rigorous methodology. Moreover, our study first conducted more comprehensive and in-depth analyses of the predictive performance of radiomics in EC DCRT response. In addition, Deantonio *et al*.^13^ included five studies and revealed the potential of 18F-FDG PET-based radiomics to predict pCR in EC NCRT. In comparison, the performance of radiomics using different imaging modalities, including PET, CECT, and MRI, were also described in our sub-analysis, all of which showed great potential in patient stratification with favorable sensitivity and specificity. However, larger sample sizes are needed to verify the results when comparing more strengths or differences among different imaging modalities.

In the era of evidence-based medicine, the only path to gaining clinical acceptability for radiomics is through rigorous study governed by rigid guidelines. Recently, Shi *et al*.^73^ presented an evaluation matrix with 13 items to evaluate the methodological quality of studies regarding EC CCRT based on RQS and TRIPOD, but they failed to perform a quantitative meta-analysis for eligible studies. Similarly, various tools, particularly IBSI items, were employed in our study to comprehensively evaluate the quality of eligible studies. Unlike their study, we scored the included studies according to each item from these appraisal tools and made a visual synthesis for their adherence rate. The RQS assessment was utilized as in other radiomic studies^23,74^. Several items in the RQS domain repeatedly lack sufficient illustration, including phantom study, cost-effectiveness, biologic correlation, and open science, suggesting that they were common issues shared by radiomic literature. Previous reviews using the TRIPOD checklist revealed that research generally followed approximately half of the TRIPOD elements^21,22^. A comparable adherence rate of TRIPOD items was demonstrated in our study, in line with other studies, which have similar problematic issues. Most studies lack details of the blindness in outcome assessments, sample size calculation, missing data processing, and model application potential. The IBSI guideline has been commonly employed for the estimation of image preprocessing steps in previous reviews^22,23,75^. Otherwise, we adapted the IBSI items to provide more normalized and deeper imaging information through adding image filters and robustness evaluation items. Although the RQS emphasizes the contribution of different scanners and vendors to radiomic feature instability, we supposed that the robustness evaluation attempts to evaluate and enhance the repeatability and reproducibility of radiomics, which deserves to be evaluated independently^76^. Nevertheless, there was no consensus within the radiomics community regarding which items must be a part of the image processing processes. To date, journals do not impose specific checklist requirements for radiomics studies, and researchers are reluctant to share the findings of their quality assessments for their radiomic studies. Among the eligible literature in our study, only three studies reported IBSI adherence^30,48,52^, and one study reported adherence to both IBSI guideline and RQS items^59^. Besides, the assessment of whether the studies followed the TRIPOD statement was only mentioned in one study^63^. Therefore, the quality assessment results should be viewed as a quality assurance of radiomic publications rather than a means of highlighting any potential flaws in the suggested model. Moreover, the ability of authors, reviewers, editors, and readers to determine if a radiomic study complies with best practices should be advocated and encouraged in the early stages of radiomic investigations. Many novel guidelines have been developed or are under development by the radiomic community, including QUADAS-AI^77^, SPRIIT-AI^78^, and CONSORT-AI^79^, which may contribute to study quality improvement.

Even though machine learning has shown great potential in a range of tasks in EC management, machine learning-based radiomic models are far from perfect. Several concerns regarding methodological issues and future applications of EC radiomics need to be carefully considered. First, the major obstacles that limit the translation of image mining studies into the clinical application are the great variability of analysis methods and the impasse to repeat the results in other tested cohorts^24^. Based on the subgroup analysis, datasets with the training cohort achieved a higher sensitivity and specificity than those with the validation cohort, which was consistent with our knowledge. However, very limited datasets from external validation were included in our study. Lack of independent validation significantly results in overestimation of the results, which could compromise the generalizability of the model. Although the definition of external validation varies between the RQS and TRIPOD statement, it is still preferable to develop EC radiomic models using independent cohorts from multiple centers in future studies^80,81^.

Second, the real clinical settings for EC patients may provide different data sources for radiomic model establishment. Since the likelihood of attaining pCR or clinical response will also be impacted by variations in radiotherapy and chemotherapy protocols, further studies are still required to determine how various therapy plans affect the predictive potential of the final models. In addition, the choice of which imaging modalities are used to establish the best model should also be considered by future researchers. Our sub-analysis indicated that the MRI-based model may provide the best predictive power. However, the results may not be reliable due to the overfitting caused by the small sample size. Moreover, there is only one study exploring the predictive power of a combined radiomic model based on both CT and 18F-FDG PET in our analysis^64^. Considering the advantages of multimodality fusion in better identification for tumor heterogeneity^82^, we suggest that multilevel multimodality fusion-based radiomics may be a good way to improve the predictive performance of models. More importantly, the best time point for imaging remains controversial. In our study, there were eleven (23.90%) studies that acquired radiomic features in images from pretreatment and post-treatment scans, and only two studies (4.30%) explored the changes between radiomic features extracted from pretreatment and mid-treatment images^43,50^. The features derived from the images during treatment rather than the baseline images could discriminate local tumor control from other confounding surroundings, such as inflammation or residual tumor bulk. Moreover, some studies have demonstrated that changes in radiomic features, which might be related to characteristics of treatment response, could outperform the volume changes for disease assessment^83^. Therefore, comprehensive analysis of multiple-time point observations may provide more information to elucidate temporal variabilities in radiomic features and enhance the potential of radiomics in EC treatment decisions. Third, the ability of radiomics to accurately predict EC patient prognosis remains a challenge. Some studies have used radiomics to predict endpoints such as OS, PFS, RFS, and DFS, but all results may be unsatisfactory, with a C-index less than 0.80. The most important issue is that the precision of follow-up cannot always be achieved because of the nature of retrospective studies. Furthermore, we noted the fact that the available EC radiomic studies are often a publication with positive results, with no studies published with a predictive AUC or C-index below 0.60. Thus, more transparency and large prospective studies are required to confirm the real clinical value of radiomics in EC management. Additionally, the algorithm choice and interpretability of radiomic features are essential aspects to improve the credibility of radiomic studies^84^. Increased DL-based radiomics has been demonstrated to perform discriminatively better than models that exclusively use handmade features. However, DL approaches frequently need more data due to their greater propensity for overfitting. Our sub-analysis observed no significant difference between ML and DL signatures, which may be attributed to limiting the advantages of DL caused by the very small sample size in DL datasets. It also brings more challenges to interpreting the significance of deep learning features when used in EC clinics. Moreover, the understanding of the relationship between radiomics and the potential tumor biology of EC is still in its early stage. At the time of writing, only four studies have conducted genomic analysis for the biological interpretability of radiomic features^48,52,53,62^. Interestingly, three of these studies were from the same research group^48,52,53^, and they identified the relationship between feature selection and genomic data from the perspective of the immune microenvironment and metabolic pathways in EC tissue. However, deeper exploration of radiogenomic models should be attempted in future investigations.

There exist some inherent limitations in this study. First, only a limited number of studies met the selection criteria for each meta-analysis, and it is important to interpret meta-analysis results with caution. Most of the included studies were acknowledged as retrospective data, although better evidence may be provided by prospective data. To provide the most credible estimation with minimum selection bias, our study included all available training, internal validation, and external validation datasets with the best discrimination model. Second, we must admit some inclusion bias due to only considering English articles, which may miss essential information from other language articles. Third, the estimation for true HR in the study may lead to the potential risk of measurement bias. Therefore, we have carefully examined the reconstructed data to ensure that all numbers of events/deaths and lengths of patient follow-up match those reported in the original publications. Fourth, considering the complexity of the development of radiomic models, several different characteristics, such as diverse ROIs, segmentation methods, and publication years, were not employed in the sub-analysis due to the small sample size. Finally, the present study evaluated only the preprocess phases of the IBSI, and several items, such as multiple imaging acquisition or phantom study from RQS, are rarely realistic for real-world practice. Therefore, a broad consensus on methods and reporting standards for radiomics research is needed to reconcile these statements.

## Conclusions

In summary, radiomic models exhibit promising potential in the prediction of chemoradiation response and prognosis in patients with EC, thus helping EC treatment decisions. Nevertheless, the insufficient quality of studies and the immature phases of the research suggest that these radiomic models are not currently available for clinical use. Thus, further prospective investigation and multicenter validation with rigid adherence to present guidelines are still needed to promote clinical translation. Moreover, persistent efforts in overcoming certain technical barriers should also be made to obtain more precise and well-designed radiomic studies.

## Ethical approval

Not applicable.

## Sources of funding

This work was supported by grants from the National Natural Science Foundation of China [Grant No. 81872699].

## Author contribution

Z.Y. and L.Z.: contributed to the conception and design of the study, the analysis and interpretation of data, and the work draft; Z.Y., J.G., and J.L.: participated in the data extraction and analysis; Z.Y., J.G., and H.S.: designed figures and tables; L.Z. and Y.P.: offered guidance in study design and revised the article critically for important intellectual content. All authors have read and approved the final version of the manuscript.

## Conflicts of interest disclosure

The authors declare that they have no financial conflicts of interest with regard to the content of this report.

## Research registration unique identifying number (UIN)

The present study has been registered on PROSPERO. ID: CRD42022371720 https://www.crd.york.ac.uk/PROSPERO/display_record.php?RecordID=371720.

## Guarantor

Lina Zhao is the guarantor of this article.

## Data availability statement

All data generated or analyzed during this study are included in this published article and its supplementary information files. All data in this manuscript is available and transparent for readers.

## Provenance and peer review

Not commissioned, externally peer-reviewed.

## Supplementary Material
