# Supplementary figures and images for: The gap before real clinical application of imaging-based machine-learning and radiomic models for chemoradiation outcome prediction in esophageal cancer: a systematic review and meta-analysis
Source: Int J Surg. 2023 Jul 17;109(8):2451–66. doi: 10.1097/JS9.0000000000000441 (PMC10442126; doi:10.1097/JS9.0000000000000441)

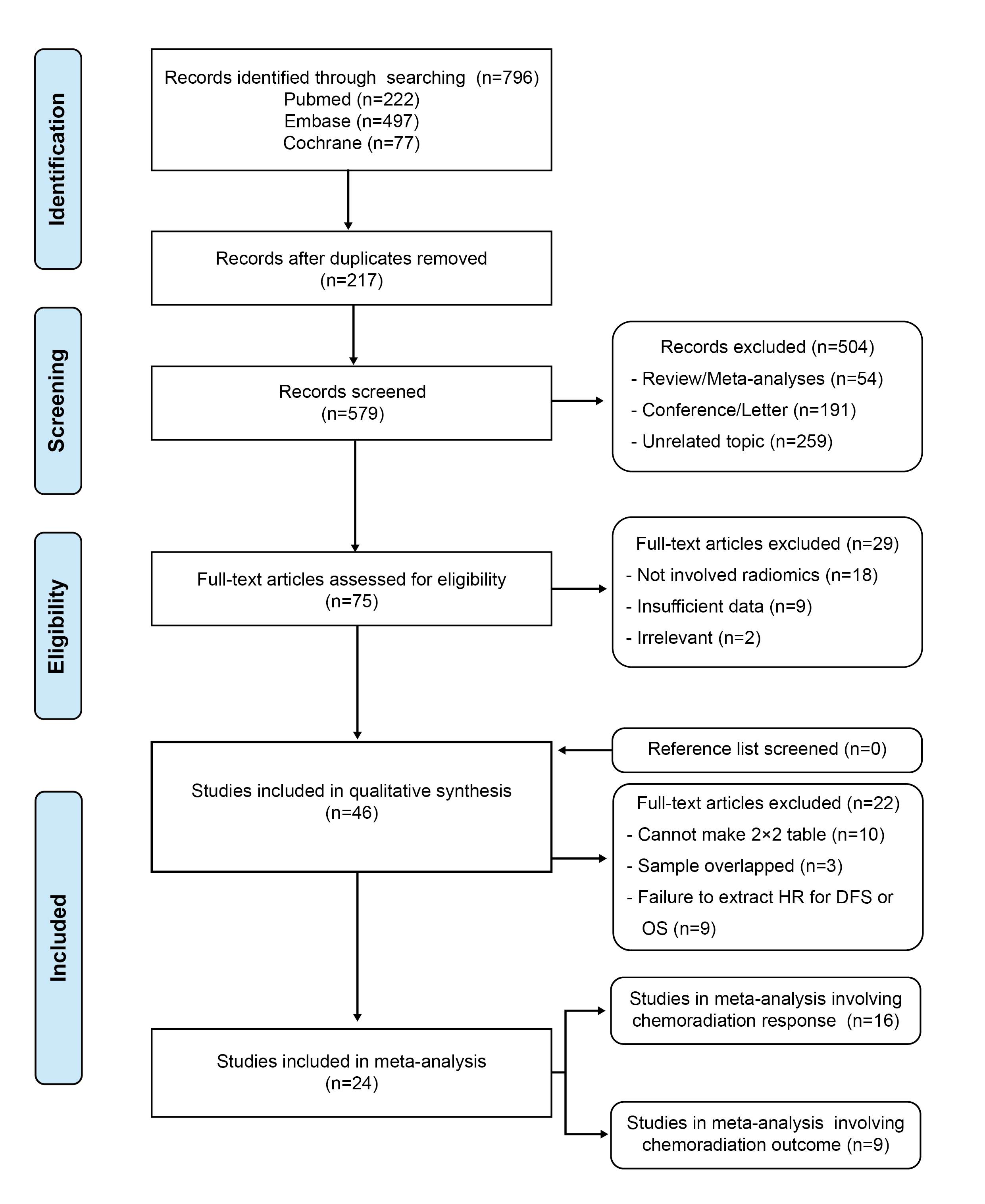

Supplement: Supplementary file 2 [file js9-109-2451-s002.tif]
