# Supplementary material for: The gap before real clinical application of imaging-based machine-learning and radiomic models for chemoradiation outcome prediction in esophageal cancer: a systematic review and meta-analysis
Source: Int J Surg. 2023 Jul 17;109(8):2451–66. doi: 10.1097/JS9.0000000000000441 (PMC10442126; doi:10.1097/JS9.0000000000000441)

**Supplementary** **materials**

**The gap before real clinical application of imaging-based machine-learning and radiomic models for chemoradiation outcome prediction in esophageal cancer: a systematic review and meta-analysis**

**List of Supplementary Materials**

Supplementary Note S1 Search strategy

Supplementary Note S2. Study eligibility and selection.

Supplementary Note S3 Data extraction sheet

Supplementary Table S1 RQS checklist elements according to six key domains

Supplementary Table S2 TRIPOD reporting completeness checklist

Supplementary Table S3 IBSI guideline reporting pre-processing steps

Supplementary Table S4 Bias risk and application concern assessment according to QUADAS-2

Supplementary Table S5 Trials classification for the proposed image mining tools development process

Supplementary Table S6 Types of prediction model studies covered by the TRIPOD statement

Supplementary Table S7 Study characteristics included in the systematic review

Supplementary Table S8 Individual RQS ratings

Supplementary Table S9 TRIPOD adherence per study

Supplementary Table S10 QUADAS assessment for each study

Supplementary Table S11 Pre-processing steps performed in each study

Supplementary Table S12 Model metrics of studies included in meta-analysis

Supplementary Table S13. Summary estimate of subgroup analysis in image-based radiomics in NCRT response prediction.

Supplementary Table S14 Subgroup analysis of radiomic utilization in comparinh CCRT OS outcome.

Supplementary Figure S1. Forest plot of DOR in EC CCRT response prediction.

Supplementary Figure S2 Forrest Plot of Pooled PLR and NLR in EC CCRT response prediction.

Supplementary Figure S3 Forest plot of pooled sensitivity and specificity in different subgroup among NCRT datasets.

Supplementary Figure S4 Forest plot of pooled sensitivity and specificity in different subgroup among DCRT datasets.

Supplementary Figure S5 Deeks funnel plot of datasets included in the first meta-analysis.

Supplementary Figure S6 Trim and fill analysis of datasets included in the first meta-analysis.

Supplementary Figure S7 Publish bias funnel plot of studies included in the second meta-analysis.

Supplementary Figure S8 Trim and fill analysis of datasets included in the second meta-analysis.

**Supplementary Note S1. Search strategy**

**1. PubMed Search Strategy**

Avalable via https://pubmed.ncbi.nlm.nih.gov

Formal search date: 31 Oct 2022

Articles retrieved: 222

#1. "Esophageal Neoplasms"[Mesh] OR (Esophageal Neoplasm[Title/Abstract]) OR (Neoplasm, Esophageal[Title/Abstract]) OR (Esophagus Neoplasm[Title/Abstract]) OR (Esophagus Neoplasms[Title/Abstract]) OR (Neoplasm, Esophagus[Title/Abstract]) OR (Neoplasms, Esophagus[Title/Abstract]) OR (Neoplasms, Esophageal[Title/Abstract]) OR (Cancer of Esophagus[Title/Abstract]) OR (Cancer of the Esophagus[Title/Abstract]) OR (Esophagus Cancer[Title/Abstract]) OR (Cancer, Esophagus[Title/Abstract]) OR (Cancers, Esophagus[Title/Abstract]) OR (Esophagus Cancers[Title/Abstract]) OR (Esophageal Cancer[Title/Abstract]) OR (Cancer, Esophageal[Title/Abstract]) OR (Cancers, Esophageal[Title/Abstract]) OR (Esophageal Cancers[Title/Abstract])

#2. (radiomic* [Title/Abstract]) OR (quantitative imaging[Title/Abstract]) OR (textur*[Title/Abstract]) OR (image analysis[Title/Abstract]) OR (radiogenomic*[Title/Abstract]) OR (histogram* [Title/Abstract])

#3."Multimodal Imaging"[Mesh] OR "Tomography, X-Ray Computed"[Mesh] OR "Positron-Emission Tomography"[Mesh] OR "Positron Emission Tomography Computed Tomography"[Mesh] OR "Magnetic Resonance Imaging"[Mesh] OR "Ultrasonography"[Mesh] OR "Diagnostic Imaging"[Mesh] OR "Radiology"[Mesh] OR((((((((((((((((PET[Title/Abstract]) OR (pet-ct[Title/Abstract])) OR (FDG[Title/Abstract])) OR (positron[Title/Abstract])) OR (emission[Title/Abstract])) OR (tomography[Title/Abstract])) OR (EUS[Title/Abstract])) OR (endoscopic[Title/Abstract])) OR (ultrasound[Title/Abstract])) OR (ultraso*[Title/Abstract])) OR (MR*[Title/Abstract])) OR (magneti*[Title/Abstract])) OR (magnetic resonance imaging[Title/Abstract])) OR (CT[Title/Abstract])) OR (computed tomography[Title/Abstract])) OR (radiology[Title/Abstract])) OR (imaging[Title/Abstract])

#4 #1 AND #2 AND #3

**2. Embase Search Strategy**

Available via www.embase.com

Formal search date: 31 Oct 2022

Articles retrieved: 497

#1. 'esophagus tumor'/exp OR 'esophageal cancers':ti,kw,ab OR 'cancers, esophageal':ti,kw,ab OR 'cancer, esophageal':ti,kw,ab OR 'esophageal cancer':ti,kw,ab OR 'esophagus cancers':ti,kw,ab OR 'cancers, esophagus':ti,kw,ab OR 'cancer, esophagus':ti,kw,ab OR 'esophagus cancer':ti,kw,ab OR 'cancer of the esophagus':ti,kw,ab OR 'cancer of esophagus':ti,kw,ab OR 'neoplasms, esophageal':ti,kw,ab OR 'neoplasms, esophagus':ti,kw,ab OR 'neoplasm, esophagus':ti,kw,ab OR 'esophagus neoplasms':ti,kw,ab OR 'esophagus neoplasm':ti,kw,ab OR 'neoplasm, esophageal':ti,kw,ab OR 'esophageal neoplasm':ti,kw,ab

#2. ‘imaging'/exp OR 'computer assisted tomography'/exp OR 'positron emission tomography'/exp OR 'positron emission tomography-computed tomography'/exp OR 'Magnetic Resonance Imaging'/exp or 'ultrasound'/exp OR 'radiology'/exp OR ‘PET’:ti,kw,ab OR ‘pet-ct’:ti,kw,ab OR ‘FDG’:ti,kw,ab OR ‘positron’:ti,kw,ab OR ‘emission’:ti,kw,ab OR ‘tomography’:ti,kw,ab OR ‘EUS’:ti,kw,ab OR ‘endoscopic’:ti,kw,ab OR ‘ultrasound’:ti,kw,ab OR ‘ultraso*’:ti,kw,ab OR ‘MR*’:ti,kw,ab OR ‘magneti*’:ti,kw,ab OR ‘magnetic resonance imaging’:ti,kw,ab OR ‘CT’:ti,kw,ab OR ‘computed tomography’:ti,kw,ab OR ‘radiology’:ti,kw,ab OR ‘imaging’:ti,kw,ab

#3 ‘radiomic*’:ti,kw,ab OR ‘quantitative imaging’:ti,kw,ab OR ‘textur*’:ti,kw,ab OR ‘image analysis’:ti,kw,ab OR ‘radiogenomic*’:ti,kw,ab OR ‘histogram*’:ti,kw,ab

#4 #1 AND #2 AND #3

**3. Cochrane**

Available via https://www.cochrane.org/

Formal search date: 31 Oct 2022

Articles retrieved: 77

#1 MeSH descriptor: [Esophageal Neoplasms] explode all trees

#2 (esophageal):ti,ab,kw OR (esophagus):ti,ab,kw OR (oesophageal):ti,ab,kw OR (oesophagus):ti,ab,kw

#3 (cancer*):ti,ab,kw OR (NEOPLAS*):ti,ab,kw OR (CARCINOMA*):ti,ab,kw OR (tumo?r*):ti,ab,kw OR (malignan*):ti,ab,kw OR (adenocarcinoma*):ti,ab,kw OR (squamous cell carcinoma*):ti,ab,kw

#4 #2 and #3

#5 #1 or #4

#6 ‘radiomic*’:ti,kw,ab OR ‘quantitative imaging’:ti,kw,ab OR ‘textur*’:ti,kw,ab OR ‘image analysis’:ti,kw,ab OR ‘radiogenomic*’:ti,kw,ab OR ‘histogram*’:ti,kw,ab

#7 MeSH descriptor: [Positron-Emission Tomography] explode all trees

#8 MeSH descriptor: [Tomography Scanners, X-Ray Computed] explode all trees

#9 MeSH descriptor: [Magnetic Resonance Imaging] explode all trees

#10 MeSH descriptor: [Ultrasonography] explode all trees

#11 MeSH descriptor: [Radiology] explode all trees

#12 ‘PET’:ti,kw,ab OR ‘pet-ct’:ti,kw,ab OR ‘FDG’:ti,kw,ab OR ‘positron’:ti,kw,ab OR ‘emission’:ti,kw,ab OR ‘tomography’:ti,kw,ab OR ‘EUS’:ti,kw,ab OR ‘endoscopic’:ti,kw,ab OR ‘ultrasound’:ti,kw,ab OR ‘ultraso*’:ti,kw,ab OR ‘MR*’:ti,kw,ab OR ‘magneti*’:ti,kw,ab OR ‘magnetic resonance imaging’:ti,kw,ab OR ‘CT’:ti,kw,ab OR ‘computed tomography’:ti,kw,ab OR ‘radiology’:ti,kw,ab OR ‘imaging’:ti,kw,ab

#13 #7 or #8 or #9 or #10 or #11 or #12

#14 #5 and #6 and #13

**Supplementary Note S2.** **Study eligibility and selection**.

**1. Studies included in** **systematic review**

***1.1 Inclusion criteria:***

1) the cohort consists of patients with histologically confirmed esophageal cancer who had received neoadjuvant chemoradiotherapy or definitive chemoradiotherapy;

2) patients had underwent at least one pre-treatment with or without post-treatment CT, MRI or PET/CT;

3) machine learning or radiomic-based quantitative image analysis for prediction of response to therapy or prognosis of patients was established.

***1.2 Exclusion*** ***criteria:***

1) duplicate studies;

2) reviews, technical reports, letters to editors, comments to published studies, conference proceedings, case reports, brief communications and articles with insufficient information for assessing the methodological quality;

3) studies are reported other than English;

4) studies in phantom or animal models

5) only included quantitative imaging features of volume, PET SUV and/or MTV, without inclusion of any additional engineered ‘radiomics’ features.

Contact with the authors was sought if the full-text version was not accessible otherwise. The reference lists of included studies were screened for additional, potentially eligible articles.

**2. Studies included in meta-analysis**

If a sufficient number of studies attempts to answer a similar question, a meta-analysis could be performed. In current study, the response and outcome prediction of esophageal cancer to CCRT were repeatedly addressed. Therefore, these studies were included in the meta-analysis.

***2.1 Inclusion criteria for meta-analysis***

1) studies with clearly defined criteria for assessing and classifying response and outcome to CCRT;

2) studies with documented sensitivity, specificity, accuracy, positive predictive value (PPV), negative predictive value (NPV), likelihood ratio, diagnostic odds ratio (DOR), or with those that could be estimated using published data.

***2.2 Exclusion criteria for meta-analysis***

1) Two-by-two contingency tables not documented, or could be calculated using published data;

2) HR and 95% CI not documented, or could be estimated using published KM curves;

3) overlapping cohorts or dataset.

**Supplementary Note S3. Data extraction sheet**

| **Field** | **Item** |
| --- | --- |
| **Bibliographical Information** | The Title of The Study |
|  | The First Authorship of The Study |
|  | Published Year |
|  | Published Journal |
|  | Impact Factor of Published Journal |
|  | Published Volume |
|  | Published Issue |
|  | Published Page |
|  | Country |
|  | Study ID, determined by First Author + Year, + Journal if needed |
| **Study Characteristics** | Study Design |
|  | Patient Condition |
|  | Patient Gender |
|  | Patient Age |
|  | Imaging Modality |
|  | Predictor |
|  | Outcome |
|  | Reference Standard |
|  | Data Splitting |
| **Radiomics Considerations** | ROI Segmentation |
|  | Radiomics Feature Extraction Details |
|  | Radiomics Feature Reduction Details |
|  | Radiomics Feature Selection Details |
|  | Selector |
| **Model Metrics** | Sample Size |
|  | Number of Events (True Positive, False Positive, False Negative, True Negative) |
|  | Sensitivity |
|  | Specificity |
|  | Accuracy |
|  | Positive Predictive Value (PPV) |
|  | Negative Predictive Value (NPV) |
|  | Positive Likelihood Ratio (PLR) |
|  | Negative Likelihood Ratio (NLR) |
|  | Diagnostic Odds Ratio (DOR) |
|  | Hazard ratio (HR) and 95% CI |

**Supplementary Table S1. RQS checklist elements according to six key domains**

| **Domain** | **No.** | **RQS scoring item** | **Points and Interpretation** |
| --- | --- | --- | --- |
| **Domain 1: Protocol quality and stability in image and segmentation (0 to 5)** | **1** | **Image protocol quality** - well-documented image protocols (for example, contrast, slice thickness, energy, etc.) and/or usage of public image protocols allow reproducibility/replicability | + 1 if protocols are well-documented  + 1 if public protocol is used |
|  | **2** | **Multiple segmentations** - possible actions are: segmentation by different physicians/algorithms/software, perturbing segmentations by (random) noise, segmentation at different breathing cycles. Analyse feature robustness to segmentation variabilities | + 1 if segmented multiple times (different physicians, algorithms, or perturbation of regions of interest) |
|  | **3** | **Phantom study on all scanners** - detect inter-scanner differences and vendor-dependent features. Analyse feature robustness to these sources of variability | + 1 if texture phantoms were used for feature robustness assessment |
|  | **4** | **Imaging at multiple time points** - collect images of individuals at additional time points. Analyse feature robustness to temporal variabilities (for example, organ movement, organ expansion/ shrinkage) | + 1 multiple time points for feature robustness assessment |
| **Domain 2: Feature selection and validation (- 8 to 8)** | **5** | **Feature reduction or adjustment for multiple testing** - decreases the risk of overfitting. Overfitting is inevitable if the number of features exceeds the number of samples. Consider feature robustness when selecting features | - 3 if neither measure is implemented  + 3 if either measure is implemented |
|  | **12** | **Validation** - the validation is performed without retraining and without adaptation of the cut-off value, provides crucial information with regard to credible clinical performance | − 5 if validation is missing  + 2 if validation is based on a dataset from the same institute/  + 3 if validation is based on a dataset from another institute/  + 4 if validation is based on two datasets from two distinct institutes/  +4 if the study validates a previously published signature/  +5 if validation is based on three or more datasets from distinct institutes  *Datasets should be of comparable size and should have at least 10 events per model feature |
| **Domain 3: Biologic/clinical validation and utility (0 to 6)** | **6** | **Multivariable analysis with non-radiomics features** (for example, EGFR mutation) - is expected to provide a more holistic model. Permits correlating/inferencing between radiomics and non-radiomics features | + 1 if multivariable analysis with non-radiomics features |
|  | **7** | **Detect and discuss biological correlates** - demonstration of phenotypic differences (possibly associated with underlying gene–protein expression patterns) deepens understanding of radiomics and biology | + 1 if present |
|  | **13** | **Comparison to ‘gold standard**’ - assess the extent to which the model agrees with/is superior to the current ‘gold standard’ method (for example, TNM-staging for survival prediction). This comparison shows the added value of radiomics | + 2 for comparison to gold standard |
|  | **14** | **Potential clinical utility** - report on the current and potential application of the model in a clinical setting (for example, decision curve analysis) | + 2 for reporting potential clinical utility |
| **Domain 4: Model performance index (0 to 5)** | **8** | **Cut-off analyses** - determine risk groups by either the median, a previously published cut-off or report a continuous risk variable. Reduces the risk of reporting overly optimistic results | + 1 if cutoff either pre-defined or at median or continuous risk variable reported |
|  | **9** | **Discrimination statistics** - report discrimination statistics (for example, C-statistic, ROC curve, AUC) and their statistical significance (for example, p-values, confidence intervals). One can also apply resampling method (for example, bootstrapping, cross-validation) | + 1 if a discrimination statistic and its statistical significance are reported  + 1 if a resampling method technique is also applied |
|  | **10** | **Calibration statistics** - report calibration statistics (for example, Calibration-in-the-large/slope, calibration plots) and their statistical significance (for example, *P*-values, confidence intervals). One can also apply resampling method (for example, bootstrapping, cross-validation) | + 1 if a calibration statistic and its statistical significance are reported  + 1 if a resampling method technique is also applied |
| **Domain 5: High level of evidence (0 to 8)** | **11** | **Prospective study registered in a trial database** - provides the highest level of evidence supporting the clinical validity and usefulness of the radiomics biomarker | + 7 for prospective validation of a radiomics signature in an appropriate trial |
|  | **15** | **Cost-effectiveness analysis** - report on the cost-effectiveness of the clinical application (for example, QALYs generated) | + 1 for cost-effectiveness analysis |
| **Domain 6: Open science and data (0 to 4)** | **16** | **Open science and data** - make code and data publicly available. Open science facilitates knowledge transfer and reproducibility of the study | + 1 if scans are open source  + 1 if region of interest segmentations are open source  + 1 if code is open source  + 1 if radiomics features are calculated on a set of representative ROIs and the calculated features and representative ROIs are open source |
| **Total points (36 = 100%)** | | | |

Note: RQS, radiomics quality score.

**Supplementary Table S2. TRIPOD reporting completeness checklist**

| **Section** | **No.** | **Item** | **Explanation** | **Values** |
| --- | --- | --- | --- | --- |
| **Title and Abstract** | **1** | **Title**-identify developing/validating a model, target population, and the outcome | #1: considered as complete if all elements of the type of study (development, validation, incremental value or combination), the target population, and outcome are included. | 0.Not documented  1. Complete |
|  | **2** | **Abstract**-provide a summary of objectives, study design, setting, participants, sample size, predictors, outcome, statistical analysis, results, and conclusions | #2 and #3b: considered as complete if ‘development’ and/or ‘validation’ is explicitly written. Synonyms instead of development such as ‘establish’, ‘build’, ‘investigate’, and ‘evaluate’ were not considered as complete. | 0. Not complete  1. Complete |
| **Introduction** | **3a** | **Background**-Explain the medical context and rationale for developing/validating the model | #3a: considered as complete if at least a simple sentence was provided to introduce the medical context and rationale for developing/validating the model. | 0. Not complete  1. Complete |
|  | **3b** | **Objective**-Specify the objectives, including whether the study describes the development/validation of the model or both. | #2 and #3b: considered as complete if ‘development’ and/or ‘validation’ is explicitly written. Synonyms instead of development such as ‘establish’, ‘build’, ‘investigate’, and ‘evaluate’ were not considered as complete | 0. Not complete  1. Complete |
| **Methods** | **4a** | **Source of data**-describe the study design or source of data (randomized trial, cohort, or registry data) | #4a: whether the study was conducted in a randomized controlled trial, cohort, or registry with a consecutive, random, or convenience series. A study was considered as complete when the terms ‘retrospective’ or ‘prospective’ were mentioned. | 0. Not documented  R. Retrospective  P. Prospective RP. Both retrospective and prospective |
|  | **4b** | **Source of data**-specify the key dates | #4b: provide the name of open-source data, or declaim that the study was performed based on institutional dataset with a specific inclusion period. | 0. Not documented  L. Local data collection  P. Public data  LP. Both local and public data |
|  | **5a** | **Participants**-specify key elements of the study setting including number and location of centers | #5a: number and location of centers should be declared in multicenter studies; monocenter study should state the location of that the study performed. | 0. Not documented  SC. Single-center data  MC. Multi-center data |
|  | **5b** | **Participants**-describe eligibility criteria for participants (inclusion and exclusion criteria) | #5b: considered as complete if a structured criterion of inclusion and exclusion were provided; only disease name was not considered as complete. | 0. Not documented  1. Documented |
|  | **5c** | **Participants**-give details of treatment received, *if relevant* | #5c: treatments are relevant in prognostic studies as they modify outcomes and relevant information should be reported. | 0. Not documented  1. Documented |
|  | **6a** | **Outcome**-clearly define the outcome, including how and when assessed | #6a: the method of assessment, e.g., histology and experience of pathologists; follow-up, frequency and modality; or expert’s opinion and experience of experts. | 0. Not defined  1. Defined either explicitly |
|  | **6b** | **Outcome**-report any actions to blind assessment of the outcome | #6b: describe weather the outcome is ideally assessed while blinded to information about the predictors. | 0. Not documented  1. Documented |
|  | **7a** | **Predictors**-clearly define all predictors, including how and when assessed | #7a: the radiomics studies involve quantitative feature extraction through an automated process; thus, the element ‘when’ was ignored. | 0. Not documented  1. Documented |
|  | **7b** | **Predictors**-report any actions to blind assessment of predictors for the outcome and other predictors | #7b: if radiomics studies were based on regions-of-interest and the blindness of readers to the reference standard was considered, they were recorded as complete. If ‘blind’ or ‘unaware of’ the reference standard was not explicitly written, it was considered as incomplete. Automatic segmentation was considered as complete. | 0. Not documented  1. Documented |
|  | **8** | **Sample size**-explain how the study size was arrived at | #8: considered as complete if the database, software or method, and results were described. | 0. Not documented  1. Documented |
|  | **9** | **Missing data**-describe how missing data were handled with details of any imputation method | #9: considered as complete if the imputation method was described when there is missing data, or how to excluded the insufficient data when imputation was not performed | 0. Not documented  E. Missing data excluded from analysis  I. Missing data included in analysis |
|  | **10a** | **Statistical analysis methods**-describe how predictors were handled | #10a: considered as complete if the statistical analysis method (e.g., t test, chi-square test) were included, and suitable for the variable type. | 0. Not documented  1. Documented |
|  | **10b** | **Statistical analysis methods**-specify type of model, all model- building procedures (any predictor selection), and method for internal validation | #10b: considered as complete if all three elements, model type (e.g., logistic regression, Cox proportional hazards model), feature selection procedure to control overfitting, and methods of internal validation (cross-validation, bootstrap sample), were included. A regularization or penalization method such as the least absolute shrinkage and selection operator (LASSO) was considered as both a feature selection procedure and internal validation, as it contains 10-fold cross-validation as a default setting. | 0. Not documented  1. Documented |
|  | **10d** | **Statistical analysis methods**-specify all measures used to assess model performance and if relevant, to compare multiple models (discrimination or calibration) | #10d: the article was considered as complete if both the discrimination and calibration index were written | 0. Not documented  1. Documented |
|  | **11** | **Risk groups**-provide details on how risk groups were created, if done | #11: considered as complete if the cutoffs were provided, e.g., disease stage, predictive absolute incidence, or risk rate. | 0. Not documented  1. Documented |
| **Results** | **13a** | **Participants-**describe the flow of participants, including the number of participants with and without the outcome. A diagram may be helpful. | #13a: considered as complete if a diagram or text description with the numbers of screened patients, excluded patients and included patients was provided. | 0. Not documented  1. Documented |
|  | **13b** | **Participants-**describe the characteristics of the participants, including the number of participants with missing data for predictors and outcome | #13b: considered as complete if a table or text description was provided. | 0. Not documented  1. Documented |
|  | **14a** | **Model development-**specify the number of participants and outcome events in each analysis | #14a: considered as complete if a table or text description was provided. | 0. Not documented  1. Documented |
|  | **14b** | **Model development-**report the unadjusted association between each candidate predictor and outcome, if done | #14b: considered as complete if the metrics and their confidence interval were provided. | 0. Not documented  1. Documented |
|  | **15a** | **Model specification-**present the full prediction model to allow predictions for individuals (regression coefficients, intercept) | #10c, #15a, and #15b: these items determine if an article describes how the obtained model predicted the outcome probabilities for an individual. If the articles described this in the methods (item 10c) and contained a full prediction model including all regression coefficients and the intercept or baseline hazard for a particular time point, they were considered as complete for item 15a. If the study contained explicit formula or a nomogram, the study was considered as complete for item 15b. | 0. Not documented  1. Documented |
|  | **15b** | **Model specification-**explain how to the use the prediction model (nomogram, calculator, etc) | #10c, #15a, and #15b: these items determine if an article describes how the obtained model predicted the outcome probabilities for an individual. If the articles described this in the methods (item 10c) and contained a full prediction model including all regression coefficients and the intercept or baseline hazard for a particular time point, they were considered as complete for item 15a. If the study contained explicit formula or a nomogram, the study was considered as complete for item 15b. | 0. Not documented  1. Documented |
|  | **16** | **Model performance-**report performance measures (with confidence intervals) for the prediction model | #16: considered as complete if the metrics (at least the discrimination outcome) and their confidence interval were provided. | 0. Reported without measure of precision  1. Reported with confidence interval or standard error |
| **Discussion** | **18** | **Limitations-**Discuss any limitations of the study | #18: considered as complete if there was a limitation paragraph, usually the paragraph before the conclusion. | 0. Not discussed  1. Discussed |
|  | **19b** | **Interpretation-**Give an overall interpretation of the results | #19b: considered as complete if there was an interpretation of results paragraph, usually the paragraph of discussion. | 0. Not documented  1. Documented |
|  | **20** | **Implications-**Discuss the potential clinical use of the model and implications for future research | #20: considered as complete if there was text description or decision curve analysis. This is different from ‘clinical validity’ in RQS criterion 14, that the decision curve analysis was necessary. | 0. Not discussed  1. Discussed |
| **Validation (types 2a, 2b, 3, and 4)** | **10c** | **Statistical analysis methods-**describe how the predictions were calculated | #10c, #15a, and #15b: these items determine if an article describes how the obtained model predicted the outcome probabilities for an individual. If the articles described this in the methods (item 10c) and contained a full prediction model including all regression coefficients and the intercept or baseline hazard for a particular time point, they were considered as complete for item 15a. If the study contained explicit formula or a nomogram, the study was considered as complete for item 15b. | 0. Not documented  1. Documented |
|  | **10e** | **Statistical analysis methods-**describe any model updating (recalibration), if done | #10e and #17: If an article describes methods to adjust (recalibrate) or update a previously developed prediction model, the article is scored. This is different from ‘comparison with gold standard’ in RQS criterion 13, in that it requires recalibration of regression coefficients and hazard ratios in the pre-existing model, and was scored if it was completely reported. | 0. Not documented  1. Documented  N/A. Not updating |
|  | **12** | **Development vs. validation-**identify any differences from the development data in setting, eligibility criteria, outcome, and predictors | #12 and #13c: considered as complete if a table comparing developing and testing dataset or text description was provided. | 0. Not documented  1. Documented |
|  | **13c** | **Participants (for validation)-**show a comparison with the development data of the distribution of important variables | #12 and #13c: considered as complete if a table comparing developing and testing dataset or text description was provided. | 0. Not documented  1. Documented |
|  | **17** | **Model updating-**report the results from any model updating, *if done* | #10e and #17: If an article describes methods to adjust (recalibrate) or update a previously developed prediction model, the article is scored. This is different from ‘comparison with gold standard’ in RQS item 13, in that it requires recalibration of regression coefficients and hazard ratios in the pre-existing model, and was scored if it was completely reported. | 0. Not documented  1. Documented  N/A. Not updating |
|  | **19a** | **Interpretation (for validation)-**discuss the results with reference to performance in the development data and any other validation data | #19a: considered as complete if there was a paragraph that discuss the influence of difference between development and validation data on the model performance. The performance of the model in the validation study should be discussed and placed in context to the model performance in the original development study and with any other existing validation studies of that model. One should highlight the main results, as well as any biases that may have affected the comparison. When the validation study shows a different (usually poorer) performance, reasons should be discussed to enhance interpretation. | 0. Not documented  1. Documented |
| **Other Information** | **21** | **Supplementary information**-provide information about the availability of supplementary resources, such as study protocol, Web calculator, and data sets | #21: considered as complete if the study provided supplementary materials and/or links for online resources, or declared that all data were provided in the manuscript. | 0. Not documented  1. Documented |
|  | **22** | **Funding-**give the source of funding and the role of the funders for the present study | #22: considered as complete if the source of funding and the role of the funders were both declared. | 0. Not documented  F. Funding source documented  FR. Funding source and role documented  NF. Stated no funding received |

Note: TRIPOD, Transparent Reporting of a multivariable prediction model for Individual Prognosis Or Diagnosis.

**Supplementary Table S3. IBSI guideline reporting pre-processing steps**

| **No. IBSI** | **Pre-Processing Steps** | **Description** |
| --- | --- | --- |
| **46** | **Intensity normalization-**describe the method and settings used to normalize intensity distributions within a patient or patient cohort. | Any kind of normalization method was accepted, such as white stripe normalization, z-score normalization, or normalization using the μ±3σ method. |
| **48** | **Segmentation method**-describe how regions of interest were segmented; describe the number of experts, their expertise and consensus strategies for manual delineation; describe methods and settings used for semi-automatic and fully automatic segmentation; describe which image was used to define segmentation in case of multi-modality imaging. | Any kind of segmentation method was accepted, such as manual segmentation, semi-automatic segmentation, or fully automatic segmentation, with or without providing number of experts, their expertise and consensus strategies for manual delineation, or settings used for semi-automatic or fully automatic segmentation. |
| **50** | **Image interpolation (iso-voxel resampling)-**describe which interpolation algorithm was used to interpolate the image; describe how the position of the interpolation grid was defined; describe how the dimensions of the interpolation grid were defined; describe how extrapolation beyond the original image was handled. | Mentioning the exact term “interpolation” or “resampling” was presumed to perform iso-voxel resampling with or without providing interpolation algorithm, the position of the interpolation grid, or how extrapolation beyond the original image was handled. |
| **56** | **Grey-level discretization-**describe the method used to discretize image intensities. | Mentioning the exact term “discretization” was presumed to perform gray-level discretization with or without providing the number of bins or the size of the bins. |
| **57** | **Image filter-**describe whether and which methods and settings were used to filter images. | Any kind of filtering method was accepted, such as Laplacian-of-Gaussian, wavelet, or a declaration of non-filtering. |
| **59** | **IBSI compliance-**state if the software used to extract the set of image biomarkers can reproduce the IBSI feature reference values. | A software is compliant if and only if it can reproduce image biomarker reference values for the digital phantom and for one or more image processing configurations using the radiomics CT phantom. We documented the name of software, and then found out whether they were IBSI compliant or not. |
| **60** | **Robustness-**describe how robustness of the image biomarkers was assessed. | Robustness is one of the key concerns for generalizability and application of radiomics models. We documented the method of robustness assessment, e.g. test-retest analysis, before the model building. |

Note: IBSI, Image Biomarker Standardization Initiative.

**Supplementary Table S4. Bias risk and application concern assessment according to QUADAS-2**

| **Domain and Description** | **Modified signaling question** | **Risk of bias** | **Applicability concern** |
| --- | --- | --- | --- |
| **Patient selection**-describe methods of patient selection: Describe included patients (prior testing, presentation, intended use of index test and setting) | Question 1: was a consecutive or random sample of patients enrolled? | Could the selection of patients have introduced bias? | Are there concerns that the included patients do not match the review question? |
|  | Question 2: was a case–control design avoided? |  |  |
|  | Question 3: did the study avoid inappropriate exclusions? |  |  |
| **Index test**-describe the index test and how it was conducted and interpreted | Question 1: were the imaging acquisition protocol, image processing approach described in detail? | Could the conduct or interpretation of the index test have introduced bias? | Are there concerns that the index test, its conduct, or interpretation differ from the review question? |
|  | Question 2: were the segmentation method(s), and feature extraction software described in detail? |  |  |
|  | Question 3: was the validation independent (i. e. external)? |  |  |
| **Reference standard-**describe the reference standard and how it was conducted and interpreted | Question: is the reference standard likely to correctly classify the target condition? | Could the reference standard, its conduct, or its interpretation have introduced bias? | Are there concerns that the target condition as defined by the reference standard does not match the review question? |
| **Flow and timing-**describe any patients who did not receive the index test(s) and/or reference standard or who were excluded from the 2x2 table (refer to flow diagram): Describe the time interval and any interventions between index test(s) and reference standard | Question: was there an appropriate interval between imaging and reference standard? | Could the patient flow have introduced bias? | -- |

Note: QUADAS-2, Quality Assessment of Diagnostic Accuracy Studies

**Supplementary Table S5. Trials classification for the proposed image mining tools development process**

| **Phase** | **Sample size** | **Type of study** | **Validation approach** | **Development stage** |
| --- | --- | --- | --- | --- |
| Discovery science | Algorithm development, phantom, simulated data | Experimental | No validation | Preclinical |
| Phase 0 | < 100 patients | Retrospective | Internal validation | Pre-marketing |
| Phase I | < 100 patients | Retrospective | Validation | Pre-marketing |
| Phase II | > 100 patients | Retrospective | Validation | Pre-marketing |
| Phase III | > 100 patients | Prospective | Validation | Pre-marketing |
| Phase IV | NA | Real-world | Monitoring | Post-marketing |

Note: The validation process may be internal (e.g., cross-validation and bootstrapping) or external (using data not used for training). The external validation may be performed using three different strategies: (i) temporal (i.e., data obtained in newly recruited patients), (ii) geographic (i.e., data collected in a different institution), and (iii) split-sample (i.e., data split from the entire dataset and kept untouched for the test).

**Supplementary Table S6. Types of prediction model studies covered by the TRIPOD Statemen**

| **Model type** | **Explanation** |
| --- | --- |
| Type 1a | Development of a prediction model where predictive performance is then directly evaluated using the same data (apparent performance). |
| Type 1b | Development of a prediction model using the entire data set, but then using resampling (e.g., bootstrapping or cross-validation) techniques to evaluate the performance and optimism of the developed model. Resampling techniques, generally referred to as “internal validation”, are recommended as a prerequisite for prediction model development, particularly if data are limited. |
| Type 2a | The data are randomly split into 2 groups: one to develop the prediction model and one to evaluate its predictive performance. This design is generally not recommended or better than type 1b, particularly in case of limited data, because it leads to lack of power during model development and validation. |
| Type 2b | The data are nonrandomly split (e.g., by location or time) into 2 groups: one to develop the prediction model and one to evaluate its predictive performance. Type 2b is a stronger design for evaluating model performance than type 2a because it allows for nonrandom variation between the 2 data sets. |
| Type 3 | Development of a prediction model using 1 data set and an evaluation of its performance on separate data (e.g., from a different study). |
| Type 4 | The evaluation of the predictive performance of an existing (published) prediction model on separate data. |

\

**Supplementary Table S7. Study characteristics included in the systematic review**

| **Study ID (Refs.)** | **Population** | **#Pat** | **Imaging modality** | **Research question** | **Phase** |
| --- | --- | --- | --- | --- | --- |
| Beukinga.2022 | Patients with locally advanced esophageal cancer (T1N1-3M0/T2–4aN0-3M0) treated with nCRT | 199 | ^18^F-FDG PET/CT×1 baseline | Evaluate the ability of a machine learning classification using baseline 18F-FDG-PET textural features to predict response to nCRT in EC patients | 0 |
| Tang.2021 | Locally advanced ESCC patients (IB-IVA) | 220 | CE-CT×1 baseline | Evaluate and validate the value of computerized 3D texture-based contrast-enhanced CT before nCRT for predicting recurrence of locally advanced ESCC within 2 years after trimodal therapy | III |
| **Murakami.2021** | **Resectable ESCC patients (IB-IV)** | **98** | **^18^F-FDG PET/CT×1 baseline** | **Assessed the value of a machine learning approach using baseline 18F-FDG-PET textural features to predict pathological complete response to chemotherapy to nCRT in ESCC patients** | **0** |
| **Ji. 2021** | **Patients with ESCC (clinical stage T1-T2,N0, M0) treated with nCRT** | **32** | **MRI (DCE)×1 baseline** | **Explore the importance of vascular permeability and texture parameters from DCE-MRI in predicting the response to nCRT in patients with ESCC** | **Discovery science** |
| Tang.2021b | Locally advanced ESCC patients (IB-IIIB) | 197 | CE-CT×1 baseline | Investigate the predictive performance of 2D and 3D image features in recurrence of ESCC patients using CT images before nCRT in radiomics studies | II |
| **Xie.2021** | **Locally advanced ESCC patients received nCRT plus surgery** | **160** | **CE-CT×2 baseline, post-treatment** | **Assessed the prognostic value on improving prognostication performance of radiomic features derived from CT performed before and after nCRT in ESCC patients** | **II** |
| **Li. 2021** | **Locally advanced ESCC patients (stage T2-4aN+/-M0) received nCRT** | **121** | **CE-CT×2 baseline, post-treatment** | **Develop a reliable approach to predict pathological response using radiomics from pre-treatment and post-treatment CT in ESCC patients** | **0** |
| **Rishi. 2021** | **Resectable cancer of the thoracic oesophagus and GEJ (IB-IIIC)** | **68** | **^18^F-FDG PET/CT×1 baseline CE-CT×1 baseline** | **Develop a radiomic-based model composited CT/PET features to predict pathological complete response and outcome following nCRT in oesophageal cancer.** | **0** |
| Beukinga.2021 | Locally advanced EC patients (T1/N1-3/M0; T2-4a/N0-3/M0) received nCRT | 96 | ^18^F-FDG PET/CT×1 baseline | Evaluate the predictive value on pathological complete response to nCRT of quantitative indices and texture features derived from 18F-FDG-PET performed before treatment in ESCC patient | 0 |
| Hu.2021 | Resectable ESCC patients (I-IVA) | 231 | CE-CT×1 baseline | Evaluate and validate the predictive performance of the CT-based model using deep learning features for predicting pathologic complete response to nCRT in ESCC | II |
| **Hu.2020** | **Resectable ESCC patients (I-IVA)** | **231** | **CE-CT×1 baseline** | **Investigate whether peritumoral radiomics features derived from baseline CT images can provide complementary predictive to enhance the ability of intratumoral radiomics to estimate pathological complete response after nCRT** | **II** |
| **Chen.2019** | **Patients with ESCC (stage I-III) treated with nCRT plus surgery** | **44** | **^18^F-FDG PET/CT×1 baseline** | **Investigate whether the quantitative indices and radiomic parameters of 18F-FDG-PET can predict event-free survival of patients with ESCC** | **I** |
| **Yang.2019** | **Locally advanced ESCC (T3-4a/N0-3) patients** | **55** | **CE-CT×1 baseline** | **Build models to predict complete pathologic response after nCRT in ESCC patients using radiomic features derived from pre-treatment CT** | **I** |
| **Larue.2018** | **Locally advanced ESCC (T1-4/N0-3/M0-M1) patients treated with nCRT plus surgery** | **239** | **CE-CT×1 baseline** | **Investigate the prognostic value of pretreatment CT radiomic features to predict 3-year overall survival of esophageal cancer patients after nCRT** | **II** |
| **Riyahi.2018** | **Patients with EC who underwent trimodality therapy (T1-4/N0-1/M0-1a)** | **20** | **^18^F-FDG PET/CT×2 baseline, post-treatment** | **Establish a framework to detect and quantify local tumor morphological changes due to nCRT using a Jacobian map and to extract quantitative radiomic features from the Jacobian map derived from PET/CT to predict the pathologic tumor response in locally advanced EC patients** | **0** |
| Beukinga.2018 | Locally advanced EC treated with nCRT (T2-4a/N0-3) | 73 | ^18^F-FDG PET/CT×2 baseline, post-treatment | Assess the value of baseline (before nCRT) and restaging (after nCRT) 18F-FDG-PET radiomics in predicting pathologic complete response to nCRT in patients with locally advanced EC | 0 |
| Beukinga.2017 | Curatively resectable EC patients who underwent nCRT (T1-4a/N0-3) | 97 | ^18^F-FDG PET/CT×1 baseline | Investigate whether constructed model based on pretreatment clinical parameters and 18F-FDG PET-derived textural features can improve predictive performance on complete response to nCRT in EC | 0 |
| Yip.2016 | Locally advanced EC treated with nCRT plus surgery (T1-3/N0-3/M0-1) | 54 | ^18^F-FDG PET/CT×2 baseline, post-treatment | Explore the relation between 18F-FDG-PET-derived textural features and quantitative indices with pathologic response and overall survival after nCRT in EC | Discovery science |
| **van Rossum.2016** | **Patients with resectable EAC who underwent nCRT followed by surgery (T2-3/N0-+)** | **217** | **^18^F-FDG PET/CT×2 baseline, post-treatment** | **Determine whether subjective and quantitative assessment of baseline and post-chemoradiation 18F-FDG-PET can improve the accuracy of predicting pathCR to nCRT in EAC beyond clinical predictors** | **0** |
| Yip. 2016b | EC patients esophageal who were treated with nCRT | 45 | ^18^F-FDG PET/CT×2 baseline, post-treatment | Investigate the usefulness of contour propagation in texture analysis using 18F-FDG-PET performed before and after nCRT for the purpose of pathologic response prediction in EC | Discovery science |
| Zhang.2014 | Patients with EC who underwent trimodality therapy (T1-4/N0-1/M0-1a) | 20 | ^18^F-FDG PET/CT×2 baseline, post-treatment | Evaluate the ability of predictive models using comprehensive tumor features from 18F-FDG-PET performed before and after nCRT for the estimation of tumor response to nCRT in patients with EC | 0 |
| Tan.2013 | Patients with EC who underwent trimodality therapy (T1-4/N0-1/M0-1a) | 20 | ^18^F-FDG PET/CT×2 baseline, post-treatment | Assess whether longitudinal patterns in 18F-FDG uptake characterized using histogram distances provide useful information for predicting the pathologic response of EC to nCRT | Discovery science |
| Tan.2013b | Patients with EC who underwent trimodality therapy (T1-4/N0-1/M0-1a) | 20 | ^18^F-FDG PET/CT×2 baseline, post-treatment | Eextract and investigate comprehensive spatial-temporal 18F-FDG-PET features before and after nCRT for the prediction of pathologic tumor response to nCRT therapy in EC | Discovery science |
| **Hirata.2020** | **Locally advanced ESCC treated with nCRT plus surgery (T1-4/N0-3)** | **58** | **MRI (DWI)×1 baseline** | **Evaluate whether pretreatment histogram derived ADC parameters can predict pathological complete response and survival in patients with ESCC after nCRT** | **Discovery science** |
| **Gong.2022** | **Localy advanced ESCC** | **397** | **CE-CT×1 baseline** | **Establish and validate a contrast-enhanced CT-based hybrid radiomics nomogram for prediction of LRFS in ESCC patients receiving CCRT in a multicenter setting** | **II** |
| Jayaprakasam.2022 | Locally advanced ESCC without distant metastases | 74 | ^18^F-FDG PET/CT×1 baseline | Assess the usefulness of radiomics features of 18F-FDG PET/CT in patients with ESCC in predicting outcomes, including cT/cN categories, PET response to induction chemotherapy, PFS, and 3 year- OS. | I |
| **An.2022** | **Locally advanced ESCC (T3/4N-/+)** | **93** | **MRI (DWI)×3 baseline, 5^th^ radiation, 10^th^ radiation** | **Investigate the association between the radiomics features extracted from tumor ADC map with response to CCRT in patients with ESCC** | **III** |
| Luo.2021 | ESCC (Stage I-IVa) | 221 | CE-CT×1 baseline | Develop a model using baseline CT textural features for predicting LPFS in ESCC patients treated with CCRT. | II |
| Kong.2021 | ESCC (Stage I-IVa) | 218 | CE-CT×1 baseline | Establish and evaluate the value of a predictive model based on enhanced CT texture features for detecting LRFS in cases of ESCC after CCRT | II |
| **Li.2021b** | **Locally**  **advanced ESCC (Stage II-IV)** | **306** | **CE-CT×1 baseline** | **Establish and validate a pretreatment CT-based deep-learning model for predicting the treatment response to CCRT among patients with ESCC** | **III** |
| Luo.2020 | ESCC (Stage I-IVa) | 226 | CE-CT×1 baseline | Develop and validate a model to predict complete response after concurrent CCRT in ESCC patients using pretreatment CT-derived shape and texture features | II |
| **Li.2020** | **Locally advanced ESCC (Stage I-IVa)** | **216** | **^18^F-FDG PET/CT×1 baseline** | **Assessed the value of baseline 18F-FDG-PET textural features on patient stratification with ESCC who do not benefit from standard CRT** | **II** |
| **Cao.2020** | **Locally advanced ESCC (Stage II-IV)** | **159** | **^18^F-FDG PET/CT×1 baseline** | **Investigate the predictive value of texture features derived from 18F-FDG-PET performed before treatment on early prediction of treatment response on thoracic ESCC treated with CCRT** | **II** |
| Xu.2020 | Locally advanced ESCC (Stage II-IV) | 134 | MRI (DCE)×1 baseline | Explore whether DCE-MRI histogram parameters can predict 12-month PFS in patients with advanced ESCC receiving CCRT | Discovery science |
| **Xie.2020** | **ESCC (Stage III)** | **57** | **CE-CT×1 baseline** | **Investigate the relationship between the tumor heterogeneity based on CT and OS in ESCC treated with CCRT** | **Discovery science** |
| Li.2019 | Locally advanced ESCC (T2-4/N0-2/M0) | 72 | MRI (DWI) ×1 baseline | Assess the prognostic value of 3D texture features based on MRI-DWI for ESCC patients undergoing CCRT | III |
| Sun.2019 | Locally advanced ESCC (T2-4/N0-3) | 72 | MRI (DWI) ×2 baseline, post-treatment | Evaluate and predict treatment response by histogram analysis of DCE-MRI to patients with locally advanced ESCC receiving CCRT | Discovery science |
| **Xie.2019** | **Locally advanced ESCC (T3-4/N0-1)** | **133** | **CT×1 baseline** | **Investigated the potential of using sub-region radiomics as a novel tumour biomarker in predicting OS of ESCC patients treated by CCRT** | **II** |
| **Jin.2019** | **EAC and ESCC (Stage I-IVa)** | **94** | **CE-CT×1 baseline** | **Explore the treatment response prediction feasibility and accuracy of an integrated model combining CT radiomic features and dosimetric parameters using machine learning for patients with EC who underwent CCRT** | **0** |
| Xiong.2018 | Locally advanced ESCC (T1-4/N0-1/M0-1) | 30 | ^18^F-FDG PET/CT×2 baseline, mid-CRT | Evaluate the predictive performance of 18F-FDG-PET-based radiomic features for local control of ESCC treated with CCRT | 0 |
| **Hou.2018** | **ESCC (T1-4/N0-2/M0-1)** | **68** | **MRI (T2W+SPAIR T2W) ×1 baseline** | **Assess the capability of histogram- and texture-based radiomic analysis using T2W and SPAIR T2W-MRI for predicting the therapeutic response of ESCC to CCRT** | **I** |
| **Hou.2017** | **ESCC (T1-4/N0-2/M0-1)** | **49** | **CE-CT×1 baseline** | **Investigate the capability of 2D and 3D image features derived from pretreatment CT using machine learning methods to predict the therapeutic response of ESCC to CCRT** | **I** |
| Paul.2017 | Locally advanced esophageal cancer | 65 | ^18^F-FDG PET/CT×1 baseline | Develop a model using GARF classifier to select radiomic features for outcome prediction in EC treated with CCRT | 0 |
| **Nakajo.2016** | **ESCC (T1-4/N0-3)** | **52** | **^18^F-FDG PET/CT×1 baseline** | **Examine whether the heterogeneity in primary tumour 18F-FDG distribution using texture analysis can predict tumour response and prognosis of patients with ESCC treated by CCRT** | **Discovery science** |
| Hatt.2013 | EAC and ESCC (Stage I-IVa) | 50 | ^18^F-FDG PET/CT×1 baseline | Assess the robustness of PET heterogeneity textural features with respect to the delineation of functional volumes and partial volume effects correction in response to CCRT | Discovery science |
| **Tixier.2011** | **EAC and ESCC (Stage I-IVb)** | **41** | **^18^F-FDG PET/CT×1 baseline** | **Develop and evaluate new parameters obtained by textural analysis of baseline PET scans for the prediction of therapy response in EC** | **Discovery science** |
|  |  |  |  |  |  |

Note:18F-FDG-PET, 18F-fluorodeoxyglucose positron emission tomography; CT, computed tomography; CE-CT, contrast-enhanced CT,; DWI diffusion-weighted image; DCE, dynamic contrast-enhanced; MRI, magnetic resonance imaging; NCRT, neoadjuvant chemoradiotherapy; ESCC, esophageal squamous cell carcinoma; EC, esophageal cancer; EAC, esophageal adenocarcinoma; RFS, relapse free survival; RQS, radiomics quality score. Bold studies included in the meta-analysis.

**Supplementary Table S8. Individual RQS ratings**

| **Study ID** | **Image Protocol** | **Multiple Segmentations** | **Phantom Study** | **Multiple Timepoints** | **Feature Reduction** | **Non Radiomics** | **Biological Correlates** | **Cut Off** | **Discrimination/ Resampling** | **Calibration/ Resampling** | **Prospective** | **Validation** | **Gold Standard** | **Clinical Utility** | **Cost** | **Open Science** |
| --- | --- | --- | --- | --- | --- | --- | --- | --- | --- | --- | --- | --- | --- | --- | --- | --- |
| Beukinga.2022 | 1 | 0 | 0 | 0 | 3 | 0 | 0 | 0 | 2 | 0 | 0 | 2 | 2 | 0 | 0 | 0 |
| Tang.2021 | 1 | 1 | 0 | 1 | 3 | 1 | 0 | 0 | 2 | 0 | 0 | 2 | 2 | 0 | 0 | 0 |
| Murakami.2021 | 1 | 1 | 0 | 0 | 3 | 0 | 0 | 0 | 2 | 0 | 0 | 2 | 2 | 0 | 0 | 0 |
| Ji. 2021 | 1 | 1 | 0 | 0 | 3 | 0 | 0 | 1 | 1 | 0 | 7 | -5 | 2 | 0 | 0 | 0 |
| Tang.2021b | 1 | 1 | 0 | 1 | 3 | 1 | 0 | 0 | 2 | 0 | 0 | 2 | 2 | 0 | 0 | 0 |
| Xie.2021 | 1 | 1 | 0 | 1 | 3 | 1 | 1 | 1 | 1 | 1 | 0 | 3 | 2 | 2 | 0 | 0 |
| Li. 2021 | 1 | 1 | 0 | 1 | 3 | 1 | 0 | 1 | 2 | 1 | 0 | 2 | 2 | 2 | 0 | 0 |
| Rishi. 2021 | 1 | 1 | 0 | 0 | -3 | 0 | 0 | 0 | 2 | 0 | 0 | 2 | 2 | 0 | 0 | 0 |
| Beukinga.2021 | 1 | 1 | 0 | 0 | -3 | 1 | 0 | 0 | 1 | 1 | 0 | -5 | 2 | 0 | 0 | 0 |
| Hu.2021 | 1 | 1 | 0 | 0 | 3 | 0 | 1 | 0 | 1 | 1 | 0 | 3 | 2 | 2 | 0 | 0 |
| Hu.2020 | 1 | 1 | 0 | 0 | 3 | 0 | 1 | 1 | 2 | 1 | 0 | 3 | 2 | 2 | 0 | 0 |
| Chen.2019 | 1 | 0 | 0 | 1 | -3 | 1 | 0 | 0 | 1 | 0 | 0 | 2 | 2 | 0 | 0 | 0 |
| Yang.2019 | 1 | 0 | 0 | 0 | 3 | 0 | 0 | 0 | 2 | 0 | 0 | 2 | 2 | 0 | 0 | 0 |
| Larue.2018 | 1 | 0 | 0 | 0 | 3 | 0 | 0 | 0 | 2 | 0 | 0 | 3 | 2 | 0 | 0 | 0 |
| Riyahi.2018 | 1 | 0 | 0 | 1 | 3 | 0 | 0 | 0 | 2 | 0 | 0 | 2 | 2 | 0 | 0 | 0 |
| Beukinga.2018 | 1 | 1 | 0 | 1 | 3 | 1 | 0 | 0 | 2 | 1 | 0 | 2 | 2 | 0 | 0 | 0 |
| Beukinga.2017 | 1 | 1 | 0 | 0 | -3 | 1 | 0 | 0 | 2 | 1 | 0 | 2 | 2 | 0 | 0 | 0 |
| Yip.2016 | 1 | 0 | 0 | 1 | -3 | 0 | 0 | 0 | 1 | 0 | 0 | -5 | 2 | 0 | 0 | 0 |
| van Rossum.2016 | 1 | 0 | 0 | 1 | -3 | 1 | 0 | 0 | 2 | 2 | 0 | 2 | 2 | 2 | 0 | 0 |
| Yip. 2016b | 1 | 0 | 0 | 1 | -3 | 0 | 0 | 0 | 1 | 0 | 0 | -5 | 2 | 0 | 0 | 0 |
| Zhang.2014 | 1 | 0 | 0 | 1 | 3 | 1 | 0 | 0 | 2 | 0 | 0 | 2 | 2 | 0 | 0 | 0 |
| Tan.2013 | 1 | 0 | 0 | 1 | -3 | 0 | 0 | 0 | 1 | 0 | 0 | -5 | 2 | 0 | 0 | 0 |
| Tan.2013b | 1 | 0 | 0 | 1 | -3 | 0 | 0 | 0 | 1 | 0 | 0 | -5 | 2 | 0 | 0 | 0 |
| Hirata.2020 | 1 | 0 | 0 | 0 | -3 | 0 | 0 | 1 | 1 | 0 | 0 | -5 | 2 | 0 | 0 | 0 |
| Gong.2022 | 1 | 1 | 0 | 0 | 3 | 1 | 0 | 1 | 1 | 1 | 0 | 4 | 2 | 0 | 0 | 0 |
| Jayaprakasam.  2022 | 1 | 1 | 0 | 0 | 3 | 0 | 0 | 0 | 2 | 0 | 0 | 2 | 2 | 0 | 0 | 0 |
| An.2022 | 1 | 0 | 0 | 1 | 3 | 0 | 0 | 0 | 1 | 0 | 7 | 3 | 2 | 0 | 0 | 0 |
| Luo.2021 | 1 | 1 | 0 | 0 | 3 | 1 | 0 | 1 | 2 | 2 | 0 | 2 | 2 | 0 | 0 | 0 |
| Kong.2021 | 1 | 0 | 0 | 1 | 3 | 1 | 0 | 1 | 2 | 2 | 0 | 2 | 2 | 2 | 0 | 0 |
| Li.2021 | 1 | 1 | 0 | 0 | 3 | 1 | 0 | 0 | 2 | 1 | 7 | 5 | 2 | 0 | 0 | 0 |
| Luo.2020 | 1 | 1 | 0 | 0 | 3 | 1 | 0 | 1 | 2 | 0 | 0 | 2 | 2 | 2 | 0 | 0 |
| Li.2020 | 1 | 1 | 0 | 0 | 3 | 1 | 0 | 1 | 0 | 0 | 0 | 3 | 2 | 2 | 0 | 0 |
| Cao.2020 | 1 | 1 | 0 | 0 | 3 | 0 | 0 | 1 | 2 | 0 | 0 | 3 | 2 | 0 | 0 | 0 |
| Xu.2020 | 1 | 1 | 0 | 0 | -3 | 0 | 0 | 1 | 0 | 0 | 0 | -5 | 2 | 0 | 0 | 0 |
| Xie.2020 | 1 | 1 | 0 | 1 | -3 | 0 | 0 | 1 | 1 | 0 | 0 | -5 | 2 | 0 | 0 | 0 |
| Li.2019 | 1 | 1 | 0 | 0 | 3 | 0 | 0 | 0 | 2 | 2 | 7 | 2 | 2 | 0 | 0 | 0 |
| Sun.2019 | 1 | 1 | 0 | 1 | -3 | 0 | 0 | 1 | 1 | 0 | 0 | -5 | 2 | 0 | 0 | 0 |
| Xie.2019 | 1 | 1 | 0 | 1 | 3 | 0 | 1 | 1 | 2 | 0 | 0 | 3 | 2 | 0 | 0 | 0 |
| Jin.2019 | 1 | 1 | 0 | 0 | 3 | 1 | 0 | 0 | 2 | 0 | 0 | 2 | 2 | 0 | 0 | 0 |
| Xiong.2018 | 1 | 1 | 0 | 1 | 3 | 0 | 0 | 1 | 2 | 0 | 7 | 2 | 2 | 0 | 0 | 0 |
| Hou.2018 | 1 | 1 | 0 | 0 | 3 | 0 | 0 | 1 | 2 | 0 | 0 | 2 | 2 | 0 | 0 | 0 |
| Hou.2017 | 1 | 1 | 0 | 1 | 3 | 0 | 0 | 1 | 2 | 0 | 0 | 2 | 2 | 0 | 0 | 0 |
| Paul.2017 | 0 | 0 | 0 | 0 | 3 | 1 | 0 | 0 | 2 | 0 | 0 | 2 | 2 | 0 | 0 | 1 |
| Nakajo.2016 | 1 | 1 | 0 | 1 | -3 | 0 | 0 | 1 | 1 | 0 | 0 | -5 | 2 | 0 | 0 | 0 |
| Hatt.2013 | 1 | 1 | 0 | 0 | -3 | 0 | 0 | 0 | 1 | 0 | 0 | -5 | 2 | 0 | 0 | 0 |
| Tixier.2011 | 1 | 0 | 0 | 0 | 3 | 0 | 0 | 0 | 1 | 0 | 0 | -5 | 2 | 0 | 0 | 0 |
| Range | 0 to 2 | 0 to 1 | 0 to 1 | 0 to 1 | -3 to 3 | 0 to 1 | 0 to 1 | 0 to 1 | 0 to 2 | 0 to 1 | 0 to 7 | -5 to 5 | 0 to 2 | 0 to 2 | 0 to 1 | 0 to 4 |

Note: RQS, radiomics quality score. Ratings are presented as ZY/JG/JL.

**Supplementary Table S9. Individual TRIPOD items score per study.**

| **Study** | **Beukinga.2022** | **Tang.2021** | **Murakami.2021** | **Ji. 2021** | **Tang.2021b** | **Xie.2021** | **Li. 2021** | **Rishi. 2021** | **Beukinga.2021** | **Hu.2021** | **Hu.2020** | **Chen.2019** | **Yang.2019** | **Larue.2018** | **Riyahi.2018** | **Beukinga.2018** | **Beukinga.2017** | **Yip.2016** | **van Rossum.2016** | **Yip. 2016b** | **Zhang.2014** | **Tan.2013** | **Tan.2013b** | **Hirata.2020** |
| --- | --- | --- | --- | --- | --- | --- | --- | --- | --- | --- | --- | --- | --- | --- | --- | --- | --- | --- | --- | --- | --- | --- | --- | --- |
| **Total** | 21- | 19 | 14 | 17 | 20 | 20 | 22 | 19 | 20 | 19 | 19 | 18 | 15 | 17 | 15 | 18 | 20 | 14 | 22 | 12 | 16 | 13 | 13 | 14 |
| **Title and Abstract** | 1 | 2 | 0 | 0 | 1 | 2 | 0 | 1 | 1 | 1 | 1 | 1 | 0 | 1 | 0 | 1 | 0 | 0 | 2 | 0 | 2 | 0 | 0 | 0 |
| **1** | 0 | 0 | 0 | 0 | 0 | 0 | 0 | 0 | 1 | 0 | 1 | 1 | 0 | 0 | 0 | 0 | 0 | 0 | 1 | 0 | 0 | 0 | 0 | 0 |
| **2** | 1 | 1 | 0 | 0 | 1 | 1 | 0 | 1 | 0 | 1 | 0 | 0 | 0 | 1 | 0 | 1 | 0 | 0 | 1 | 0 | 1 | 0 | 0 | 0 |
| **Introduction** | 1 | 1 | 1 | 2 | 2 | 1 | 2 | 2 | 1 | 2 | 2 | 1 | 1 | 2 | 2 | 1 | 2 | 1 | 2 | 1 | 1 | 1 | 1 | 1 |
| **3a** | 1 | 1 | 1 | 1 | 1 | 1 | 1 | 1 | 1 | 1 | 1 | 1 | 1 | 1 | 1 | 1 | 1 | 1 | 1 | 1 | 1 | 1 | 1 | 1 |
| **3b** | 0 | 0 | 0 | 1 | 1 | 0 | 1 | 1 | 0 | 1 | 1 | 0 | 0 | 1 | 1 | 0 | 1 | 0 | 1 | 0 | 0 | 0 | 0 | 0 |
| **Methods** | 10 | 9 | 7 | 9 | 9 | 9 | 10 | 7 | 10 | 9 | 8 | 8 | 7 | 7 | 7 | 9 | 9 | 6 | 9 | 6 | 7 | 6 | 6 | 7 |
| **4a** | R | R | R | P | R | R | R | R | R | R | R | R | R | R | R | R | R | R | R | R | R | R | R | R |
| **4b** | L | L | L | L | L | L | L | L | L | L | L | L | L | L | L | L | L | L | L | L | L | L | L | L |
| **5a** | SC | SC | SC | SC | SC | MC | SC | SC | SC | MC | MC | SC | SC | MC | SC | SC | SC | SC | SC | SC | SC | SC | SC | SC |
| **5b** | 1 | 1 | 1 | 1 | 1 | 1 | 1 | 1 | 1 | 1 | 1 | 1 | 0 | 1 | 0 | 1 | 0 | 0 | 1 | 0 | 0 | 0 | 0 | 0 |
| **5c, if relevant** | 1 | 0 | 1 | 1 | 0 | 1 | 1 | 1 | 1 | 1 | 1 | 1 | 1 | 0 | 0 | 1 | 1 | 1 | 1 | 1 | 1 | 1 | 1 | 1 |
| **6a** | 1 | 1 | 0 | 1 | 1 | 1 | 1 | 1 | 1 | 0 | 0 | 1 | 1 | 0 | 1 | 1 | 1 | 1 | 1 | 1 | 1 | 1 | 1 | 1 |
| **6b** | 1 | 0 | 0 | 0 | 0 | 0 | 0 | 0 | 0 | 0 | 0 | 0 | 0 | 0 | 0 | 0 | 0 | 0 | 0 | 0 | 0 | 0 | 0 | 0 |
| **7a** | 1 | 1 | 1 | 1 | 1 | 1 | 1 | 1 | 1 | 1 | 0 | 1 | 1 | 0 | 1 | 0 | 1 | 1 | 1 | 1 | 1 | 1 | 1 | 1 |
| **7b** | 0 | 1 | 0 | 1 | 1 | 0 | 1 | 0 | 1 | 1 | 1 | 0 | 0 | 0 | 0 | 0 | 0 | 0 | 0 | 0 | 0 | 0 | 0 | 1 |
| **8** | 0 | 0 | 0 | 0 | 0 | 0 | 0 | 0 | 0 | 0 | 0 | 0 | 0 | 1 | 0 | 0 | 1 | 0 | 0 | 0 | 0 | 0 | 0 | 0 |
| **9** | E | 0 | 0 | 0 | 0 | 0 | 0 | 0 | I | 0 | 0 | I | 0 | 0 | 0 | E | E | 0 | I | 0 | 0 | 0 | 0 | 0 |
| **10a** | 1 | 1 | 1 | 1 | 1 | 1 | 1 | 1 | 1 | 1 | 1 | 1 | 1 | 1 | 1 | 1 | 1 | 1 | 1 | 1 | 1 | 1 | 1 | 1 |
| **10b** | 1 | 1 | 1 | 1 | 1 | 1 | 1 | 0 | 0 | 1 | 1 | 0 | 1 | 1 | 1 | 1 | 0 | 0 | 0 | 0 | 1 | 0 | 0 | 0 |
| **10d** | 0 | 0 | 0 | 0 | 0 | 1 | 1 | 0 | 1 | 1 | 1 | 0 | 0 | 0 | 0 | 1 | 1 | 0 | 1 | 0 | 0 | 0 | 0 | 0 |
| **11, if done** | 0 | 0 | 0 | 1 | 0 | 1 | 0 | 0 | 0 | 0 | 1 | 0 | 0 | 0 | 0 | 0 | 0 | 0 | 0 | 0 | 0 | 0 | 0 | 1 |
| **Results** | 5 | 4 | 2 | 2 | 4 | 3 | 6 | 4 | 3 | 2 | 3 | 4 | 3 | 2 | 2 | 3 | 5 | 3 | 5 | 2 | 2 | 3 | 2 | 4 |
| **13a** | 1 | 1 | 0 | 0 | 1 | 0 | 1 | 0 | 0 | 0 | 0 | 0 | 0 | 0 | 0 | 0 | 1 | 0 | 1 | 0 | 0 | 0 | 0 | 0 |
| **13b** | 1 | 1 | 1 | 0 | 1 | 1 | 1 | 1 | 1 | 1 | 1 | 1 | 1 | 1 | 0 | 1 | 1 | 0 | 1 | 0 | 1 | 0 | 1 | 1 |
| **14a** | 1 | 1 | 0 | 0 | 1 | 0 | 1 | 1 | 1 | 0 | 1 | 1 | 0 | 0 | 0 | 0 | 1 | 1 | 1 | 1 | 0 | 1 | 0 | 1 |
| **14b, if done** | 0 | 0 | 0 | 1 | 0 | 0 | 1 | 0 | 0 | 0 | 0 | 0 | 1 | 1 | 1 | 1 | 0 | 0 | 1 | 0 | 1 | 0 | 0 | 1 |
| **15a** | 1 | 0 | 0 | 1 | 0 | 0 | 1 | 1 | 0 | 0 | 0 | 1 | 1 | 0 | 1 | 1 | 1 | 1 | 1 | 0 | 0 | 1 | 0 | 1 |
| **15b** | 0 | 0 | 0 | 0 | 0 | 1 | 1 | 0 | 0 | 0 | 0 | 0 | 0 | 0 | 0 | 0 | 0 | 0 | 0 | 0 | 0 | 0 | 0 | 0 |
| **16** | 1 | 1 | 1 | 1 | 1 | 1 | 1 | 1 | 1 | 1 | 1 | 1 | 1 | 1 | 1 | 1 | 1 | 1 | 1 | 1 | 1 | 1 | 1 | 1 |
| **Discussion** | 3 | 3 | 3 | 3 | 3 | 3 | 3 | 3 | 3 | 3 | 3 | 3 | 2 | 3 | 3 | 3 | 3 | 3 | 3 | 2 | 3 | 3 | 3 | 2 |
| **18** | 1 | 1 | 1 | 1 | 1 | 1 | 1 | 1 | 1 | 1 | 1 | 1 | 1 | 1 | 1 | 1 | 1 | 1 | 1 | 0 | 1 | 1 | 1 | 1 |
| **19b** | 1 | 1 | 1 | 1 | 1 | 1 | 1 | 1 | 1 | 1 | 1 | 1 | 1 | 1 | 1 | 1 | 1 | 1 | 1 | 1 | 1 | 1 | 1 | 1 |
| **20** | 1 | 1 | 1 | 1 | 1 | 1 | 1 | 1 | 1 | 1 | 1 | 1 | 0 | 1 | 1 | 1 | 1 | 1 | 1 | 1 | 1 | 1 | 1 | 0 |
| **Other information** | 1 | 0 | 1 | 1 | 1 | 2 | 1 | 2 | 2 | 2 | 2 | 1 | 2 | 2 | 1 | 1 | 1 | 1 | 1 | 1 | 1 | 0 | 1 | 0 |
| **21** | 1 | 0 | 0 | 1 | 0 | 1 | 1 | 1 | 1 | 1 | 1 | 1 | 1 | 1 | 1 | 1 | 1 | 0 | 1 | 1 | 0 | 0 | 0 | 0 |
| **22** | NF | 0 | FR | F | FR | FR | F | NF | NF | FR | FR | 0 | NF | FR | F | 0 | 0 | FR | F | 0 | FR | F | FR | 0 |
| **Validation*** | N/A | 3 | 2 | N/A | 3 | 4 | N/A | N/A | N/A | 3 | 4 | 2 | 3 | 4 | N/A | N/A | N/A | N/A | N/A | N/A | N/A | N/A | N/A | N/A |
| **Model type** | 1b | 2a | 1b | 1a | 2a | 3 | 1b | 1b | 1b | 3 | 3 | 2a | 2a | 3 | 1b | 1b | 1b | 1a | 1b | 1a | 1b | 1a | 1a | 1a |
| **10c** | N/A | 1 | 1 | N/A | 1 | 1 | N/A | N/A | N/A | 1 | 1 | 1 | 1 | 1 | N/A | N/A | N/A | N/A | N/A | N/A | N/A | N/A | N/A | N/A |
| **10e, if done** | N/A | N/A | N/A | N/A | N/A | N/A | N/A | N/A | N/A | N/A | N/A | N/A | N/A | N/A | N/A | N/A | N/A | N/A | N/A | N/A | N/A | N/A | N/A | N/A |
| **12** | N/A | 1 | 1 | N/A | 1 | 1 | N/A | N/A | N/A | 0 | 1 | 1 | 1 | 1 | N/A | N/A | N/A | N/A | N/A | N/A | N/A | N/A | N/A | N/A |
| **13c** | N/A | 0 | 0 | N/A | 0 | 1 | N/A | N/A | N/A | 1 | 1 | 0 | 1 | 1 | N/A | N/A | N/A | N/A | N/A | N/A | N/A | N/A | N/A | N/A |
| **17, if done** | N/A | N/A | N/A | N/A | N/A | N/A | N/A | N/A | N/A | N/A | N/A | N/A | N/A | N/A | N/A | N/A | N/A | N/A | N/A | N/A | N/A | N/A | N/A | N/A |
| **19a** | N/A | 1 | 0 | N/A | 1 | 1 | N/A | N/A | N/A | 1 | 1 | 0 | 0 | 1 | N/A | N/A | N/A | N/A | N/A | N/A | N/A | N/A | N/A | N/A |

**Supplementary Table S9. Individual TRIPOD items score per study (continued).**

| **Study** | **Gong.2022** | **Jayaprakasam.2022** | **An.2022** | **Luo.2021** | **Kong.2021** | **Li.2021b** | **Luo.2020** | **Li.2020** | **Cao.2020** | **Xu.2020** | **Xie.2020** | **Li.2019** | **Sun.2019** | **Xie.2019** | **Jin.2019** | **Xiong.2018** | **Hou.2018** | **Hou.2017** | **Paul.2017** | **Nakajo.2016** | **Hatt.2013** | **Tixier.2011** |
| --- | --- | --- | --- | --- | --- | --- | --- | --- | --- | --- | --- | --- | --- | --- | --- | --- | --- | --- | --- | --- | --- | --- |
| **Total** | 22 | 19 | 19 | 24 | 19 | 19 | 21 | 18 | 18 | 14 | 15 | 17 | 15 | 19 | 15 | 17 | 16 | 16 | 14 | 17 | 12 | 14 |
| **Title and Abstract** | 2 | 0 | 0 | 2 | 0 | 2 | 1 | 0 | 1 | 0 | 0 | 0 | 0 | 0 | 0 | 0 | 0 | 0 | 0 | 0 | 0 | 0 |
| **1** | 1 | 0 | 0 | 1 | 0 | 1 | 0 | 0 | 1 | 0 | 0 | 0 | 0 | 0 | 0 | 0 | 0 | 0 | 0 | 0 | 0 | 0 |
| **2** | 1 | 0 | 0 | 1 | 0 | 1 | 1 | 0 | 0 | 0 | 0 | 0 | 0 | 0 | 0 | 0 | 0 | 0 | 0 | 0 | 0 | 0 |
| **Introduction** | 2 | 1 | 2 | 2 | 1 | 2 | 2 | 1 | 2 | 1 | 1 | 1 | 1 | 2 | 1 | 1 | 1 | 1 | 1 | 1 | 1 | 1 |
| **3a** | 1 | 1 | 1 | 1 | 1 | 1 | 1 | 1 | 1 | 1 | 1 | 1 | 1 | 1 | 1 | 1 | 1 | 1 | 1 | 1 | 1 | 1 |
| **3b** | 1 | 0 | 1 | 1 | 0 | 1 | 1 | 0 | 1 | 0 | 0 | 0 | 0 | 1 | 0 | 0 | 0 | 0 | 0 | 0 | 0 | 0 |
| **Methods** | 8 | 10 | 9 | 9 | 9 | 10 | 8 | 8 | 7 | 6 | 6 | 8 | 6 | 8 | 7 | 8 | 8 | 7 | 7 | 7 | 6 | 7 |
| **4a** | R | R | P | R | R | P | R | R | R | R | R | P | R | R | R | R | R | R | R | R | R | R |
| **4b** | L | L | L | L | L | L | L | L | L | L | L | L | L | L | L | L | L | L | L | L | L | L |
| **5a** | MC | SC | MC | SC | SC | MC | SC | MC | MC | SC | MC | SC | SC | MC | SC | SC | SC | SC | SC | SC | SC | SC |
| **5b** | 1 | 1 | 1 | 1 | 1 | 1 | 1 | 1 | 1 | 1 | 0 | 1 | 1 | 1 | 0 | 1 | 0 | 0 | 0 | 0 | 0 | 0 |
| **5c, if relevant** | 1 | 0 | 1 | 1 | 1 | 1 | 1 | 1 | 1 | 1 | 1 | 1 | 1 | 1 | 1 | 1 | 1 | 1 | 0 | 1 | 1 | 1 |
| **6a** | 1 | 1 | 1 | 1 | 1 | 1 | 1 | 1 | 1 | 1 | 1 | 0 | 1 | 1 | 1 | 1 | 1 | 1 | 1 | 1 | 1 | 1 |
| **6b** | 0 | 0 | 0 | 0 | 0 | 1 | 0 | 0 | 0 | 0 | 0 | 0 | 0 | 0 | 0 | 0 | 0 | 0 | 0 | 0 | 0 | 0 |
| **7a** | 0 | 1 | 1 | 1 | 1 | 1 | 1 | 1 | 0 | 0 | 1 | 1 | 0 | 1 | 1 | 1 | 1 | 0 | 1 | 1 | 1 | 1 |
| **7b** | 0 | 1 | 1 | 0 | 0 | 0 | 0 | 0 | 0 | 0 | 0 | 0 | 0 | 0 | 0 | 0 | 0 | 0 | 0 | 1 | 0 | 0 |
| **8** | 0 | 0 | 0 | 0 | 0 | 0 | 0 | 0 | 0 | 0 | 0 | 0 | 0 | 0 | 0 | 0 | 1 | 1 | 0 | 0 | 0 | 0 |
| **9** | 0 | E | 0 | 0 | 0 | 0 | 0 | 0 | 0 | 0 | 0 | 0 | 0 | 0 | 0 | 0 | 0 | 0 | 0 | 0 | 0 | 0 |
| **10a** | 1 | 1 | 1 | 1 | 1 | 1 | 1 | 1 | 1 | 1 | 1 | 1 | 1 | 1 | 1 | 1 | 1 | 1 | 1 | 1 | 1 | 1 |
| **10b** | 1 | 1 | 1 | 1 | 1 | 1 | 1 | 1 | 1 | 0 | 0 | 1 | 0 | 1 | 1 | 1 | 1 | 1 | 1 | 0 | 0 | 1 |
| **10d** | 1 | 0 | 0 | 1 | 1 | 1 | 0 | 0 | 0 | 0 | 0 | 1 | 0 | 0 | 0 | 0 | 0 | 0 | 0 | 0 | 0 | 0 |
| **11, if done** | 1 | 0 | 0 | 1 | 1 | 0 | 1 | 1 | 1 | 1 | 1 | 0 | 1 | 1 | 0 | 1 | 1 | 1 | 0 | 1 | 0 | 0 |
| **Results** | 6 | 3 | 4 | 6 | 4 | 1 | 5 | 4 | 4 | 3 | 4 | 4 | 4 | 4 | 4 | 3 | 3 | 3 | 2 | 5 | 3 | 3 |
| **13a** | 1 | 1 | 1 | 1 | 0 | 0 | 1 | 0 | 0 | 0 | 0 | 0 | 0 | 0 | 1 | 0 | 0 | 0 | 0 | 1 | 0 | 1 |
| **13b** | 1 | 1 | 1 | 1 | 1 | 1 | 1 | 1 | 1 | 1 | 1 | 1 | 1 | 1 | 1 | 1 | 1 | 1 | 0 | 1 | 1 | 1 |
| **14a** | 1 | 0 | 1 | 1 | 0 | 0 | 1 | 1 | 1 | 1 | 1 | 1 | 1 | 1 | 1 | 0 | 1 | 1 | 0 | 1 | 0 | 0 |
| **14b, if done** | 0 | 0 | 0 | 1 | 0 | 0 | 1 | 1 | 1 | 1 | 1 | 1 | 1 | 0 | 0 | 0 | 1 | 1 | 1 | 1 | 0 | 1 |
| **15a** | 1 | 0 | 0 | 1 | 1 | 0 | 0 | 1 | 1 | 1 | 1 | 1 | 1 | 0 | 0 | 1 | 0 | 0 | 1 | 1 | 1 | 0 |
| **15b** | 1 | 0 | 0 | 1 | 1 | 0 | 1 | 0 | 0 | 0 | 0 | 0 | 0 | 1 | 0 | 0 | 0 | 0 | 0 | 0 | 0 | 0 |
| **16** | 1 | 1 | 1 | 1 | 1 | 0 | 1 | 1 | 1 | 0 | 1 | 1 | 1 | 1 | 1 | 1 | 1 | 1 | 1 | 1 | 1 | 1 |
| **Discussion** | 3 | 3 | 3 | 3 | 3 | 3 | 3 | 3 | 3 | 3 | 3 | 3 | 3 | 3 | 3 | 3 | 3 | 3 | 3 | 3 | 2 | 3 |
| **18** | 1 | 1 | 1 | 1 | 1 | 1 | 1 | 1 | 1 | 1 | 1 | 1 | 1 | 1 | 1 | 1 | 1 | 1 | 1 | 1 | 1 | 1 |
| **19b** | 1 | 1 | 1 | 1 | 1 | 1 | 1 | 1 | 1 | 1 | 1 | 1 | 1 | 1 | 1 | 1 | 1 | 1 | 1 | 1 | 1 | 1 |
| **20** | 1 | 1 | 1 | 1 | 1 | 1 | 1 | 1 | 1 | 1 | 1 | 1 | 1 | 1 | 1 | 1 | 1 | 1 | 1 | 1 | 0 | 1 |
| **Other information** | 1 | 2 | 1 | 2 | 2 | 1 | 2 | 2 | 1 | 1 | 1 | 1 | 1 | 2 | 0 | 2 | 1 | 2 | 1 | 1 | 0 | 0 |
| **21** | 1 | 1 | 1 | 1 | 1 | 1 | 1 | 1 | 1 | 0 | 0 | 1 | 0 | 1 | 0 | 1 | 1 | 1 | 0 | 1 | 0 | 0 |
| **22** | F | FR | F | NF | FR | 0 | NF | FR | 0 | NF | FR | F | NF | FR | F | FR | F | NF | FR | 0 | 0 | 0 |
| **Validation*** | 4 | 3 | 3 | 4 | 4 | 3 | 4 | 2 | 3 | N/A | N/A | N/A | N/A | 3 | N/A | N/A | 2 | 3 | N/A | N/A | N/A | N/A |
| **Model type** | 3 | 2a | 3 | 2a | 2a | 3 | 2a | 3 | 3 | 1a | 1a | 1b | 1a | 3 | 1b | 1b | 2a | 2a | 1b | 1a | 1a | 1a |
| **10c** | 1 | 1 | 1 | 1 | 1 | 1 | 1 | 1 | 1 | N/A | N/A | N/A | N/A | 1 | N/A | N/A | 1 | 1 | N/A | N/A | N/A | N/A |
| **10e, if done** | N/A | N/A | N/A | N/A | N/A | N/A | N/A | N/A | N/A | N/A | N/A | N/A | N/A | N/A | N/A | N/A | N/A | N/A | N/A | N/A | N/A | N/A |
| **12** | 1 | 1 | 1 | 1 | 1 | 1 | 1 | 0 | 0 | N/A | N/A | N/A | N/A | 0 | N/A | N/A | 0 | 0 | N/A | N/A | N/A | N/A |
| **13c** | 1 | 0 | 1 | 1 | 1 | 1 | 1 | 0 | 1 | N/A | N/A | N/A | N/A | 1 | N/A | N/A | 1 | 1 | N/A | N/A | N/A | N/A |
| **17, if done** | N/A | N/A | N/A | N/A | N/A | N/A | N/A | N/A | N/A | N/A | N/A | N/A | N/A | N/A | N/A | N/A | N/A | N/A | N/A | N/A | N/A | N/A |
| **19a** | 1 | 1 | 0 | 1 | 1 | 0 | 1 | 1 | 1 | N/A | N/A | N/A | N/A | 1 | N/A | N/A | 0 | 1 | N/A | N/A | N/A | N/A |

**Note:** 1=reported, 0=not reported. The reported individual items were considered as the basic adherence of guideline.

**Supplementary Table S10. QUADAS assessment for each study**

| **Study ID** | **Risk of Bias** | | | | **Applicability Concern** | | |
| --- | --- | --- | --- | --- | --- | --- | --- |
|  | **Patient Selection** | **Index Test** | **Reference Standard** | **Flow and Timing** | **Patient Selection** | **Index Test** | **Reference Standard** |
| Beukinga.2022 | ☺ | ☺ | ? | ☺ | ☺ | ☺ | ☹ |
| Tang.2021 | ☺ | ☺ | ☺ | ? | ☺ | ☺ | ☺ |
| Murakami.2021 | ? | ☺ | ☺ | ☺ | ? | ☺ | ☺ |
| Ji. 2021 | ☺ | ☹ | ☺ | ☺ | ☺ | ☹ | ☺ |
| Tang.2021b | ☺ | ☺ | ☺ | ☺ | ☺ | ☺ | ☺ |
| Xie.2021 | ☺ | ☺ | ☺ | ? | ☺ | ☺ | ☺ |
| Li. 2021 | ☺ | ☺ | ☺ | ☺ | ☺ | ☺ | ☺ |
| Rishi. 2021 | ☺ | ☺ | ☺ | ☺ | ☺ | ☺ | ☺ |
| Beukinga.2021 | ☺ | ? | ☺ | ☺ | ☺ | ? | ☺ |
| Hu.2021 | ☺ | ☺ | ☺ | ☺ | ☺ | ☺ | ☺ |
| Hu.2020 | ☺ | ☺ | ☺ | ☺ | ☺ | ☺ | ☺ |
| Chen.2019 | ☺ | ☺ | ☺ | ☹ | ☺ | ☺ | ☺ |
| Yang.2019 | ☺ | ☺ | ☺ | ☺ | ☺ | ☺ | ☺ |
| Larue.2018 | ☺ | ☺ | ☺ | ☺ | ☺ | ☺ | ☺ |
| Riyahi.2018 | ? | ☺ | ? | ☺ | ? | ☺ | ? |
| Beukinga.2018 | ☺ | ☺ | ☺ | ☺ | ☺ | ☺ | ☺ |
| Beukinga.2017 | ☺ | ? | ☺ | ☺ | ☺ | ? | ☺ |
| Yip.2016 | ? | ☹ | ? | ☺ | ? | ☹ | ? |
| van Rossum.2016 | ☺ | ☺ | ☺ | ☺ | ☺ | ☺ | ☺ |
| Yip. 2016b | ? | ☹ | ? | ☺ | ? | ? | ? |
| Zhang.2014 | ? | ☺ | ☺ | ☺ | ? | ☺ | ☺ |
| Tan.2013 | ☺ | ☹ | ? | ☺ | ☺ | ☹ | ? |
| Tan.2013b | ☺ | ☹ | ? | ☺ | ☺ | ☹ | ? |
| Hirata.2020 | ☺ | ☹ | ☺ | ☺ | ☺ | ☹ | ☺ |
| Gong.2022 | ☺ | ☺ | ? | ☺ | ☺ | ☺ | ? |
| Jayaprakasam.2022 | ☺ | ? | ☹ | ☺ | ☺ | ☺ | ☹ |
| An.2022 | ? | ☺ | ☺ | ☺ | ? | ☺ | ☺ |
| Luo.2021 | ☺ | ? | ☺ | ☺ | ☺ | ? | ☺ |
| Kong.2021 | ☺ | ☺ | ☺ | ☺ | ☺ | ☺ | ☺ |
| Li.2021 | ☺ | ☺ | ☺ | ☺ | ☺ | ☺ | ☺ |
| Luo.2020 | ☺ | ? | ☺ | ☺ | ☺ | ☺ | ☺ |
| Li.2020 | ? | ☺ | ☺ | ? | ☺ | ☺ | ☺ |
| Cao.2020 | ☺ | ☺ | ☺ | ☺ | ☺ | ☺ | ☺ |
| Xu.2020 | ☺ | ? | ? | ? | ☺ | ? | ☹ |
| Xie.2020 | ☺ | ? | ☺ | ? | ☺ | ☺ | ☺ |
| Li.2019 | ? | ☺ | ☺ | ? | ? | ? | ☺ |
| Sun.2019 | ☺ | ☺ | ☺ | ☺ | ☺ | ☺ | ☺ |
| Xie.2019 | ☺ | ? | ☺ | ☺ | ☺ | ? | ☺ |
| Jin.2019 | ☺ | ☺ | ☺ | ? | ☺ | ☺ | ☺ |
| Xiong.2018 | ☺ | ☺ | ☺ | ☺ | ? | ☺ | ☺ |
| Hou.2018 | ? | ? | ☺ | ☺ | ? | ? | ☺ |
| Hou.2017 | ? | ? | ☺ | ☺ | ? | ? | ☺ |
| Paul.2017 | ? | ☺ | ☺ | ☹ | ? | ? | ☺ |
| Nakajo.2016 | ☺ | ? | ☺ | ? | ☺ | ☺ | ☺ |
| Hatt.2013 | ☺ | ? | ☺ | ☺ | ? | ☺ | ☺ |
| Tixier.2011 | ☺ | ? | ☺ | ☺ | ☺ | ☺ | ☺ |
|  |  |  |  |  |  |  |  |

Note: ☹, high; ☺, low; ?, unclear. QUADAS, quality assessment diagnostic accuracy studies.

**Supplementary Table S11. Pre-processing steps performed in each study**

| **Study ID** | **Intensity normalization** | **Segmentation method** | **Image interpolation/iso-voxel resampling** | **Grey-level discretization** | **Image filtering** | **IBSI compliance of radiomics extraction software** | **Robustness of imaging biomarkers assessment** |
| --- | --- | --- | --- | --- | --- | --- | --- |
| Beukinga.2022 | Y | Y | Y | N | Y | N | N |
| Tang.2021 | Y | Y | N | N | N | Y | Y |
| Murakami.2021 | N | Y | N | N | Y | Y | N |
| Ji. 2021 | N | Y | N | N | N | N | N |
| Tang.2021b | Y | Y | N | N | N | Y | Y |
| Xie.2021 | N | Y | N | N | Y | Y | Y |
| Li. 2021 | N | Y | N | N | N | Y | Y |
| Rishi. 2021 | Y | Y | N | Y | Y | N | N |
| Beukinga.2021 | N | Y | Y | Y | Y | N | N |
| Hu.2021 | Y | Y | N | Y | Y | Y | Y |
| Hu.2020 | Y | Y | Y | N | Y | Y | Y |
| Chen.2019 | N | Y | Y | N | N | N | N |
| Yang.2019 | Y | Y | N | N | Y | Y | N |
| Larue.2018 | N | N | Y | N | Y | N | N |
| Riyahi.2018 | N | N | N | N | Y | N | N |
| Beukinga.2018 | N | Y | Y | Y | Y | N | Y |
| Beukinga.2017 | Y | Y | N | Y | N | N | Y |
| Yip.2016 | N | Y | Y | N | N | N | N |
| van Rossum.2016 | N | Y | N | N | N | Y | Y |
| Yip. 2016b | N | Y | Y | N | Y | N | N |
| Zhang.2014 | N | Y | N | N | N | N | N |
| Tan.2013 | Y | Y | N | N | N | Y | N |
| Tan.2013b | Y | Y | N | N | Y | N | N |
| Hirata.2020 | N | Y | N | N | N | N | N |
| Gong.2022 | Y | Y | N | N | N | Y | Y |
| Jayaprakasam.2022 | N | Y | Y | N | N | N | N |
| An.2022 | N | Y | N | N | N | Y | N |
| Luo.2021 | Y | Y | Y | N | Y | Y | N |
| Kong.2021 | N | Y | N | N | N | Y | N |
| Li.2021 | Y | Y | N | N | N | N | Y |
| Luo.2020 | Y | Y | N | N | Y | Y | Y |
| Li.2020 | Y | Y | N | N | Y | Y | N |
| Cao.2020 | Y | Y | Y | N | N | Y | N |
| Xu.2020 | N | Y | N | N | N | N | N |
| Xie.2020 | N | N | N | N | Y | Y | N |
| Li.2019 | Y | Y | Y | N | N | N | N |
| Sun.2019 | N | Y | N | N | N | N | N |
| Xie.2019 | N | Y | N | Y | N | N | N |
| Jin.2019 | N | N | N | N | N | N | N |
| Xiong.2018 | N | Y | N | N | Y | N | N |
| Hou.2018 | Y | Y | Y | Y | Y | Y | N |
| Hou.2017 | N | Y | Y | Y | Y | N | Y |
| Paul.2017 | N | N | Y | N | N | N | N |
| Nakajo.2016 | N | Y | N | N | N | N | N |
| Hatt.2013 | N | N | Y | N | Y | N | Y |
| Tixier.2011 | Y | Y | Y | N | N | N | N |

Note: Y=Yes, reported; N=No, not reported.

**Supplementary Table S12.** **Model metrics of studies included in meta-analysis**

| **Study** | **Year** | **Dataset** | **TP** | **FP** | **TN** | **FN** | **Total** | **AUC** | **Imaging modality** | **Predictor** | **Algorithm model** | **Feature** |
| --- | --- | --- | --- | --- | --- | --- | --- | --- | --- | --- | --- | --- |
| **NCRT response pCR vs. non-pCR** | | | | | | | | | | | | |
| Murakami et al | 2021 | Internal validation | 10 | 2 | 13 | 1 | 26 | 0.95 | PET | Radiomics | NN | Texture |
| Li et al | 2021 | Internal validation | 43 | 15 | 55 | 8 | 121 | 0.84 | CECT | Radiomics+clinical | LR | Morphology+First Order+Texture |
| Rishi et al | 2021 | Internal validation | 26 | 8 | 26 | 8 | 68 | 0.87 | PET | Radiomics | LR | First Order+Texture |
| Hu et al | 2020 | Training | 61 | 13 | 74 | 13 | 161 | 0.91 | CECT | Radiomics | SVM | First Order+Texture |
| Hu et al | 2020 | External validation | 28 | 8 | 31 | 3 | 70 | 0.85 | CECT | Radiomics | SVM | First Order+Texture |
| Yang et al | 2019 | Training | 17 | 5 | 20 | 2 | 44 | 0.86 | CECT | Radiomics | LR LASSO regression | Morphology+First Order+Texture |
| Yang et al | 2019 | Internal validation | 4 | 2 | 5 | 0 | 55 | 0.79 | CECT | Radiomics | LR LASSO regression | Morphology+First Order+Texture |
| van Rossum et al | 2016 | Internal validation | 39 | 27 | 131 | 20 | 217 | 0.77 | PET | Radiomics+clinical | LR | Morphology+First Order+Texture |
| Ji et al | 2021 | Training | 15 | 1 | 15 | 1 | 32 | 0.98 | MRI | Radiomics+MRI parameters | LR | Texture |
| Riyahi et al | 2018 | Internal validation | 8 | 1 | 10 | 1 | 20 | 0.94 | PET | Radiomics | SVM | First Order+Texture |
| Hirata et al | 2020 | Training | 8 | 6 | 42 | 2 | 58 | 0.86 | MRI | Radiomics | NA | First Order |
|  |  |  |  |  |  |  |  |  |  |  |  |  |
| **DCRT response vs. non-response** | | | | | | | | | | | | |
| An et al | 2022 | Training | 36 | 3 | 14 | 0 | 23 | 0.82 | MRI | Radiomics+clinical | SVM | First Order+Texture |
| An et al | 2022 | Internal validation | 16 | 2 | 3 | 2 | 53 | 0.74 | MRI | Radiomics | SVM | First Order+Texture |
| An et al | 2022 | Extnernal validation | 9 | 1 | 5 | 2 | 17 | 0.84 | MRI | Radiomics+clinical | SVM | First Order+Texture |
| Li et al | 2021 | Training | 118 | 1 | 41 | 43 | 203 | 0.91 | CECT | Radiomics+clinical | CNN | DL feature |
| Li et al | 2021 | Extnernal validation | 65 | 0 | 14 | 24 | 103 | 0.86 | CECT | Radiomics+clinical | CNN | DL feature |
| Cao et al | 2020 | Training | 55 | 1 | 20 | 17 | 93 | 0.84 | PET | Radiomics | LR | First Order+Texture |
| Cao et al | 2020 | Extnernal validation | 42 | 2 | 10 | 12 | 66 | 0.84 | PET | Radiomics | LR | First Order+Texture |
| Jin et al | 2019 | Internal validation | 31 | 5 | 31 | 27 | 94 | 0.69 | CECT | Radiomics + dosimetric parameters | XGBoost | First Order+Texture |
| Hou et al | 2018 | Training | 21 | 1 | 19 | 2 | 43 | 0.93 | MRI | Radiomics | SVM | First Order+Texture+DL |
| Hou et al | 2018 | Internal validation | 11 | 2 | 9 | 3 | 25 | 0.84 | MRI | Radiomics | ANN | First Order+Texture+DL |
| Hou et al | 2017 | Training | 25 | 1 | 10 | 1 | 37 | 0.93 | CECT | Radiomics | ANN | First Order+Texture+DL |
| Hou et al | 2017 | Internal validation | 6 | 1 | 4 | 1 | 12 | 0.80 | CECT | Radiomics | ANN | First Order+Texture+DL |
| Tixier et al | 2011 | Training | 26 | 3 | 8 | 4 | 41 | 0.89 | PET | Radiomics | NA | First Order+Texture |
| An et al | 2022 | Training | 36 | 3 | 14 | 0 | 23 | 0.82 | MRI | Radiomics+clinical | SVM | First Order+Texture |

Note: NCRT, neoadjuvant chemoradiotherapy; DCRT, definitive chemoradiation; pCR, pathologic complete response; DL, deep learning; LR, logistic regression; NN, neural networks; SVM, support vector machines; NA, not available.

**Supplementary Table S13.** **Summary estimate of subgroup analysis in image-based radiomics in NCRT response prediction.**

|  | **No. of dataset** | **Sensitivity** | **Heterogeneity** | | **Specificity** | **Heterogeneity** | |
| --- | --- | --- | --- | --- | --- | --- | --- |
|  |  |  | **P value*** | **I^2^** |  | **P value** | **I^2^** |
| **Overrall** | 11 | 0.83 (0.76-0.89) | 0.1 | 37.70% | 0.83 (0.79-0.86) | 0.82 | 0.00% |
| **Sample** |  |  |  |  |  |  |  |
| <100 | 8 | 0.88 (0.81-0.94) | 0.77 | 0.00% | 0.84 (0.78-0.89) | 0.73 | 0.00% |
| ≥100 | 3 | 0.78 (0.66-0.88) | -- | -- | 0.83 (0.78-0.87) | -- | -- |
| **Cohort** |  |  |  |  |  |  |  |
| Train | 4 | 0.86 (0.78-0.92) | 0.67 | 0.00% | 0.86 (0.81-0.91) | 0.70 | 0.00% |
| Validation | 7 | 0.83 (0.74-0.91) | 0.10 | 43.55% | 0.82 (0.78-0.86) | 0.87 | 0.00% |
| **Classifier** |  |  |  |  |  |  |  |
| LR | 6 | 0.83 (0.73-0.91) | 0.06 | 51.92% | 0.82 (0.78-0.87) | 0.63 | 0.00% |
| SVM | 3 | 0.86 (0.79-0.92) | -- | -- | 0.85 (0.78-0.91) | -- | -- |
| **Imaging modality** |  |  |  |  |  |  |  |
| CECT | 5 | 0.87 (0.81-0.92) | 0.80 | 0.00% | 0.82 (0.76-0.87) | 0.75 | 0.00% |
| PET | 4 | 0.76 (0.65-0.86) | 0.25 | 27.38% | 0.84 (0.78-0.89) | 0.74 | 0.00% |
| MRI | 2 | 0.89 (0.73-0.99) | -- | -- | 0.90 (0.80-0.96) | -- | -- |
| **Combine model** |  |  |  |  |  |  |  |
| Only radiomic | 8 | 0.86 (0.80-0.91) | 0.82 | 0.00% | 0.84 (0.79-0.89) | 0.83 | 0.00% |
| radiomic+clinical | 3 | 0.81 (0.64-0.94) | -- | -- | 0.83 (0.78-0.88) | -- | -- |
| **Feature** |  |  |  |  |  |  |  |
| Morphology+first order+texture | 4 | 0.83 (0.68-0.94) | 0.05 | 62.19% | 0.82 (0.77-0.87) | 0.73 | 0.00% |
| First order+texture | 4 | 0.84 (0.77-0.90) | 0.54 | 0.00% | 0.83 (0.77-0.89) | 0.61 | 0.00% |
| Only first order or texture | 3 | 0.90 (0.77-0.99) | -- | -- | 0.89 (0.81-0.96) | -- | -- |

Note: * P-Value for heterogeneity within each subgroup.

**Supplementary Table S14. Subgroup analysis of radiomic utilization in comparing CCRT OS outcome.**

|  | **No. of dataset** | **Pooled HR** | | | **P value**^#^ |
| --- | --- | --- | --- | --- | --- |
|  |  | **HR (95CI)** | **P value*** | **I^2^** |  |
| **Overrall** | 13 | 2.49 (1.91-3.25) | <0.05 | 55.8% |  |
| **Sample** |  |  |  |  | 0.78 |
| <100 | 9 | 2.33 (1.90-2.87) | <0.05 | 62.3% |  |
| ≥100 | 4 | 2.18 (1.70-2.79) | 0.13 | 47.5% |  |
| **Cohort** |  |  |  |  | 0.83 |
| Train | 7 | 2.49 (1.92-3.24) | 0.08 | 47.1% |  |
| Validation | 6 | 2.15 (1.76-2.62) | <0.05 | 66.7% |  |
| **Feature** |  |  |  |  | 0.34 |
| Radiomic features | 10 | 2.16 (1.77-2.64) | <0.05 | 60.3% |  |
| DL features | 3 | 2.47 (1.89-3.21) | 0.15 | 48.2% |  |
| **Imaging modality** |  |  |  |  | 0.22 |
| CECT | 8 | 2.17 (1.84-2.56) | 0.09 | 43.9% |  |
| PET | 5 | 3.67 (2.13-6.34) | <0.05 | 64.8% |  |
| **Treatment** |  |  |  |  | 0.79 |
| NCRT | 3 | 1.85 (1.29-2.64) | 0.33 | 9.2% |  |
| DCRT | 10 | 2.38 (2.00-2.85) | <0.05 | 61.5% |  |

Note: * P-Value for heterogeneity within each subgroup; # P-Value for heterogeneity within meta-regression between subgroups.

**Supplementary Figure S1. Forest plot of the effect size calculated as DOR for datasets investigating the diagnostic accuracy of radiomics in chemoradiotherapy response prediction in EC patients.** A. The DOR for predicting NCRT. B. The DOR for predicting DCRT. NCRT, neoadjuvant chemoradiotherapy; DCRT, definitive chemoradiation; TP number of good responders (or pCR) correctly diagnosed, FN number of good responders (or pCR) diagnosed as poor (or non-pCR), FP number of poor responders (or non-pCR) diagnosed as good (or pCR), TN number of poor responders (or non-pCR) correctly diagnosed.

**
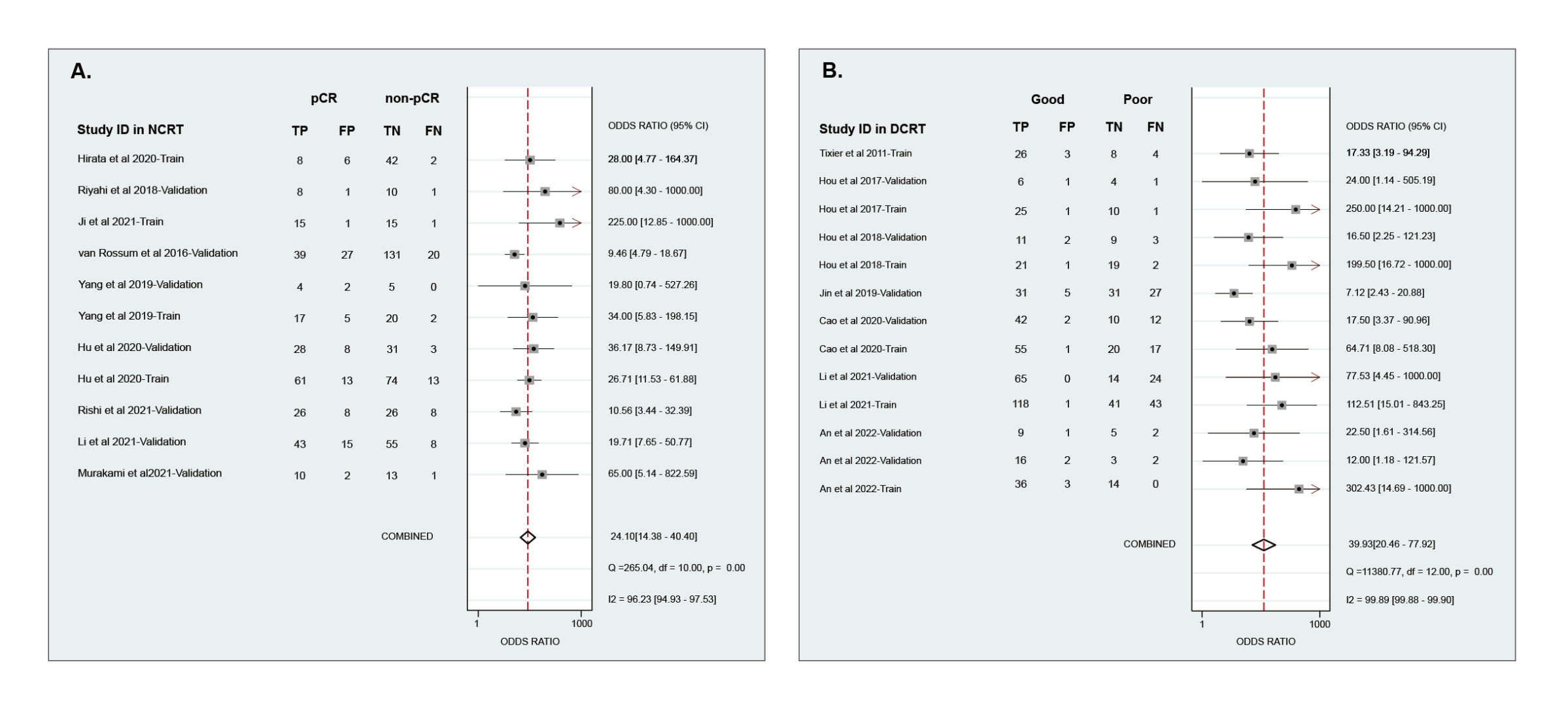
**

**Supplementary Figure S2. Forrest Plot of Pooled PLR and NLR in EC CCRT response prediction.** (A) Positive likelihood ratio in NCRT, (B) Negative likelihood ratio in NCRT, (C) Positive likelihood ratio in DCRT, (D) Negative likelihood ratio in DCRT. The numbers are pooled estimates with 95% CIs in parentheses; horizontal lines indicate 95% Cis; pooled result for all studies is presented as a black diamond.


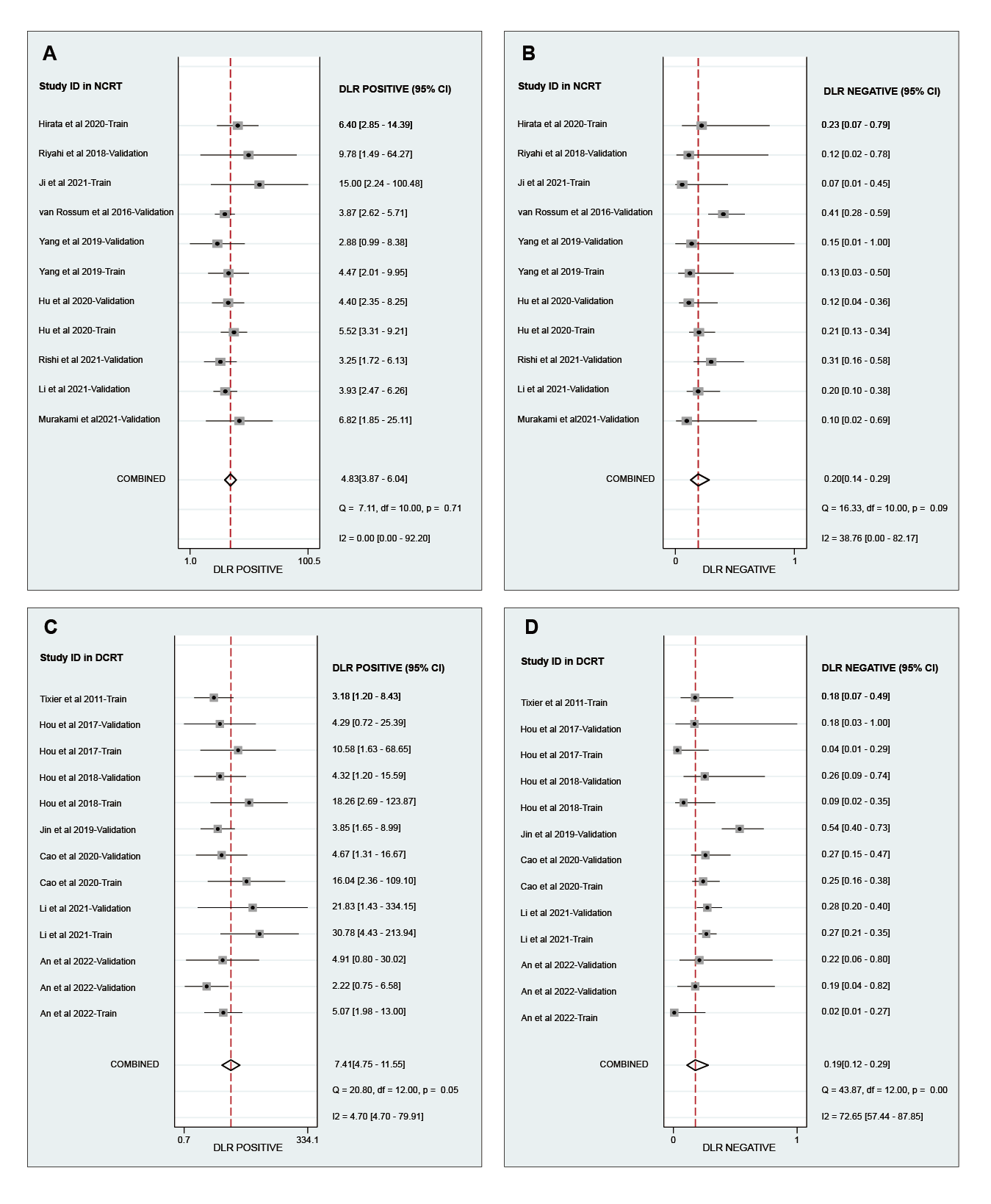


**Supplementary Figure S3. Forest plot of pooled sensitivity and specificity in different subgroup among DCRT datasets.** (A) sample sizes (<100 vs. ≥100), (B) training set vs. validation set, (C) radiomic vs deep learning features, (D) imaging modalities (CECT vs. PET, vs. MRI), (E) only radiomic model vs. combination with clinical information.


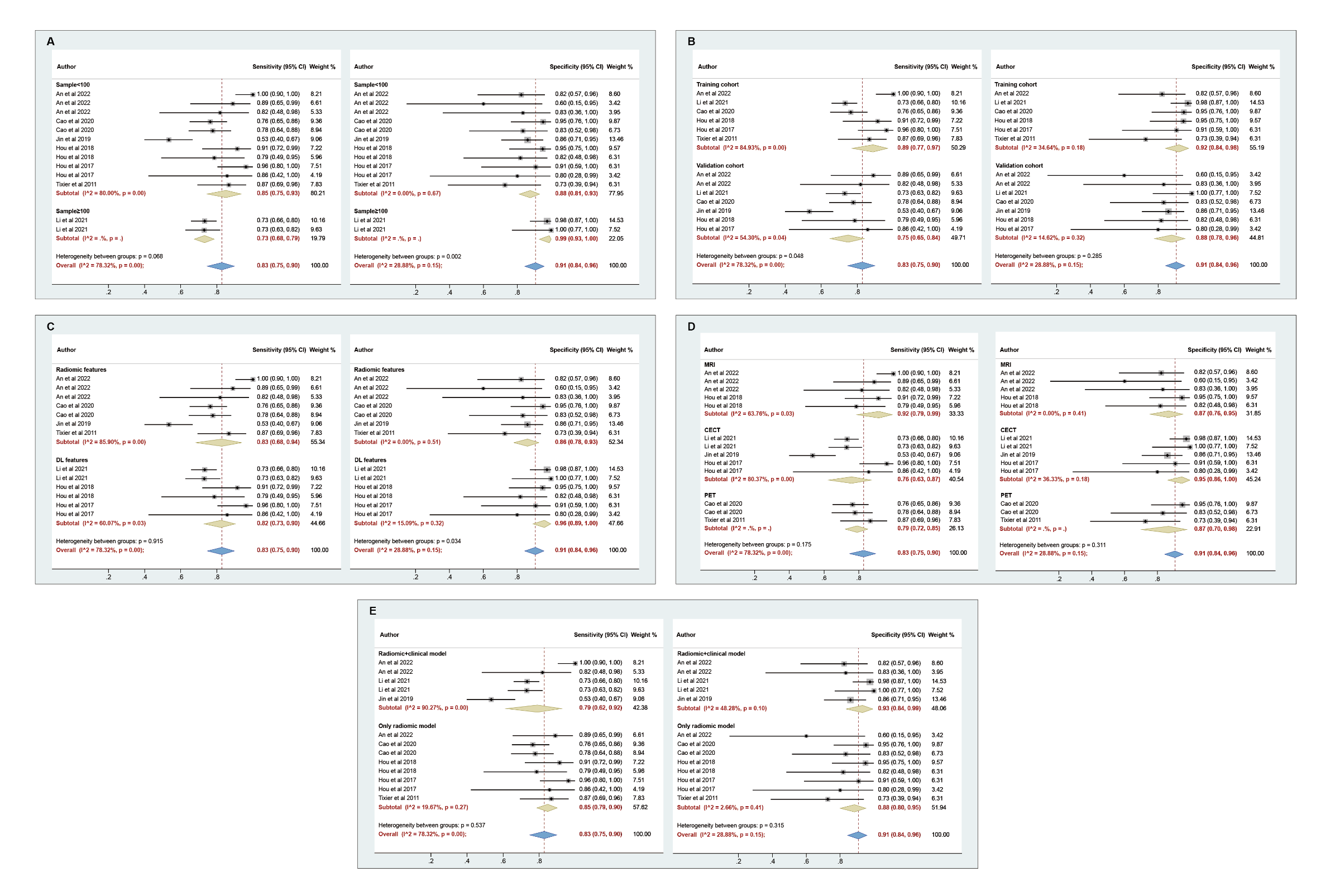


**Supplementary Figure S4. Forest plot of pooled sensitivity and specificity in different subgroup among NCRT datasets.** (A) sample sizes (<100 vs. ≥100), (B) training set vs. validation set, (C) model classifier with LR vs. SVM, (D) imaging modalities (CECT vs. PET, vs. MRI), (E) only radiomic model vs. combination with clinical information, (F) different radiomic features (morphology+first order+texture vs. others).


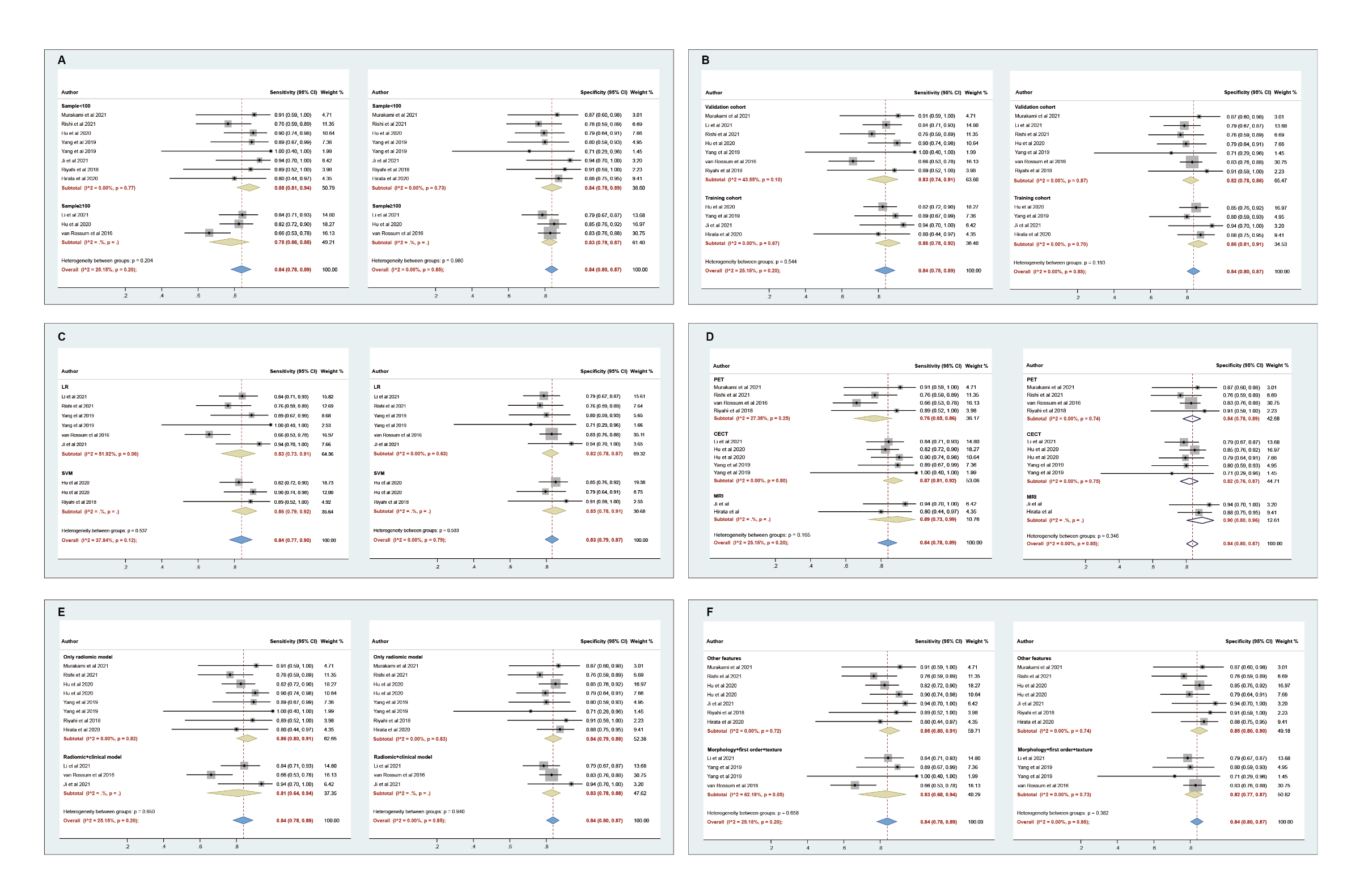


**Supplementary Figure S5. Deeks funnel plot of datasets included in the first meta-analysis.** The Deeks funnel plot asymmetry test revealed that the likelihood of publication bias was low in (A) NCRT datasets (p=0.06) and (B) DCRT datasets (p=0.58).


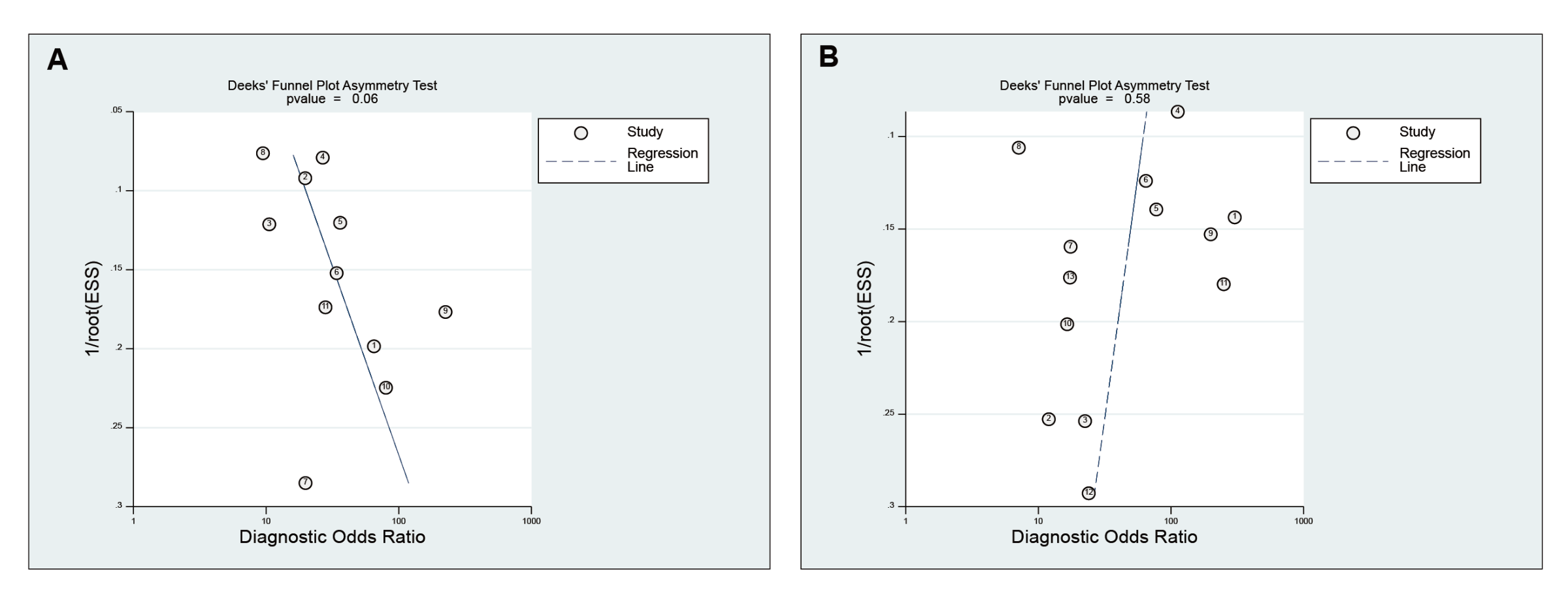


**Supplementary Figure S6. Trim and fill analysis of datasets included in the first meta-analysis.** Although the trim and fill analysis showed the missing datasets in (A) NCRT datasets and (B) DCRT datasets, the adjusted predictive performance was still statistically significant.
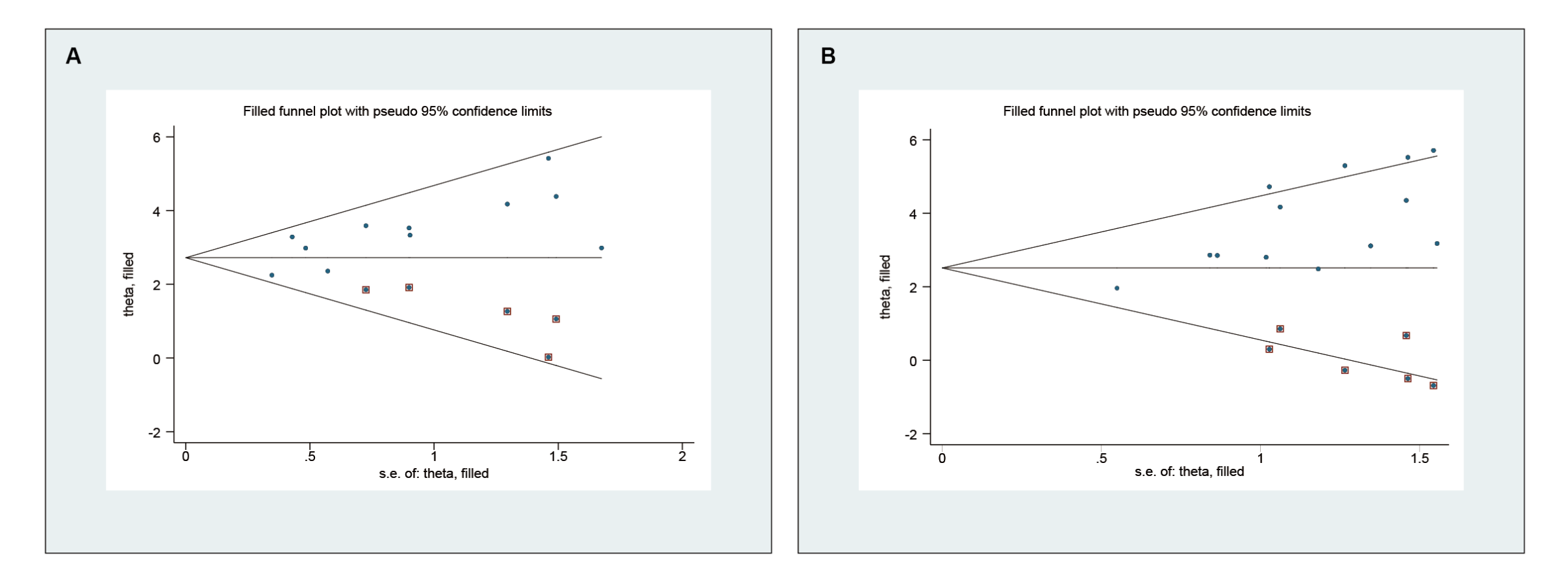


**Supplementary Figure S7. Publish bias funnel plot of studies included in the second meta-analysis.** The likelihood of publication bias was relatively low in (A) DFS datasets (p=0.620 in Egger’s test; 0.452 in Begg’s test) and (B) OS datasets (p=0.052 in Egger’s test; 0.100 in Begg’s test).


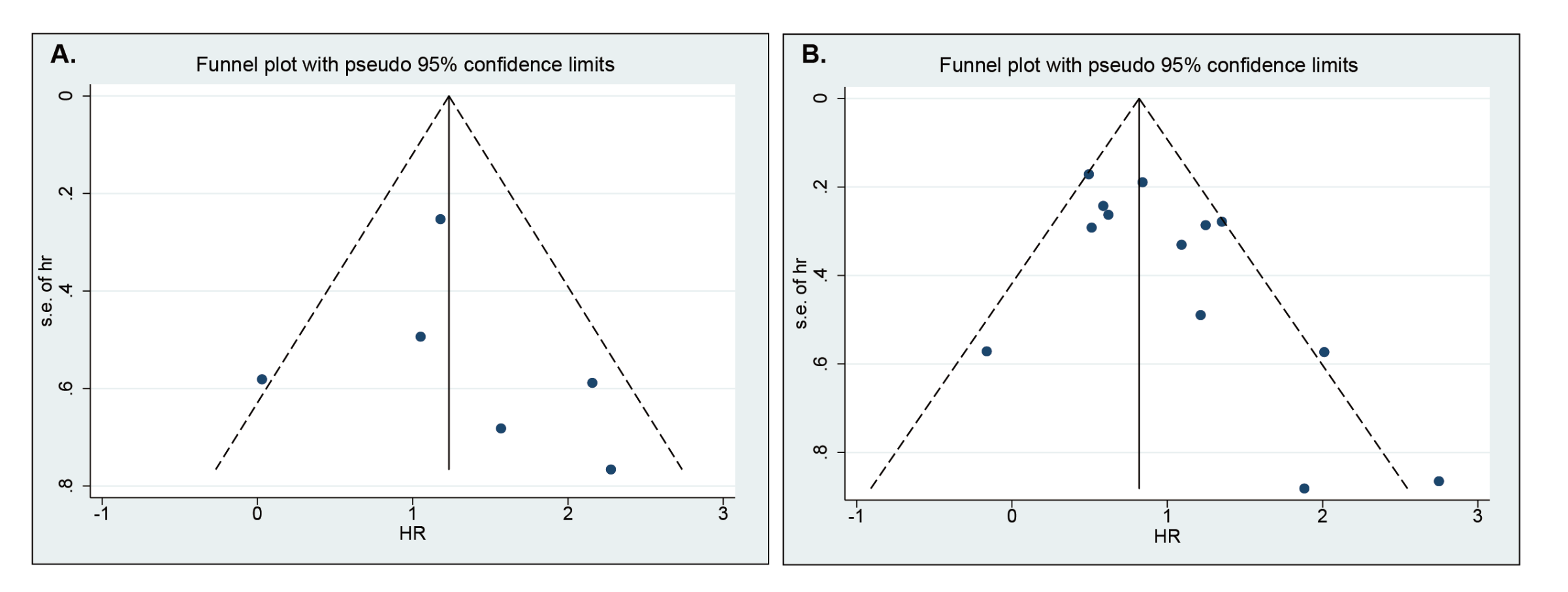


**Supplementary Figure S8. Trim and fill analysis of datasets included in the second meta-analysis.** Although the trim and fill analysis showed the missing datasets in (A) DFS datasets and (B) OS datasets, the adjusted predictive performance was still statistically significant.


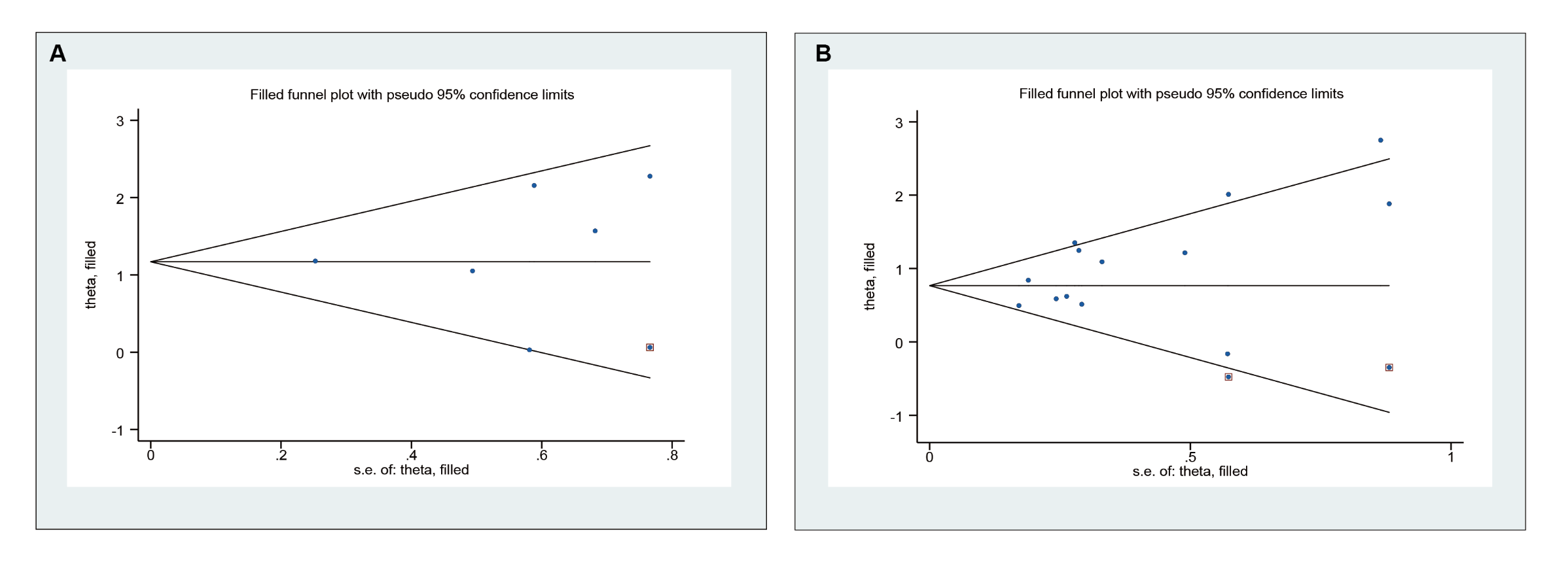

Supplement: Supplementary file 4 [file js9-109-2451-s004.docx]
